# Deletion of hepatic growth hormone receptor contributes to the pathogenesis of lean MAFLD by elevating CD36

**DOI:** 10.1093/lifemeta/loaf037

**Published:** 2025-10-28

**Authors:** Dong Yu, Xiao Yang, Xiaonan Zhang, Xiaoxin Wang, Zicheng Pu, Ai Mi, Liyuan Ran, Fang Zhang, Bin Liang, Yingjie Wu

**Affiliations:** Institute of Genome Engineered Animal Models for Human Diseases, National Center of Genetically Engineered Animal Models for International Research, Liaoning Province Key Lab of Genome Engineered Animal Models, Dalian Medical University, Dalian, Liaoning 116044, China; Key Laboratory of Endocrine Glucose & Lipids Metabolism and Brain Aging, Ministry of Education, Shandong Provincial Hospital, Science and Technology Innovation Center, Shandong First Medical University & Shandong Academy of Medical Sciences, Jinan, Shandong 250021, China; School of Life Sciences, Shandong First Medical University & Shandong Academy of Medical Sciences, Tai’an, Shandong 271016, China; Institute of Genome Engineered Animal Models for Human Diseases, National Center of Genetically Engineered Animal Models for International Research, Liaoning Province Key Lab of Genome Engineered Animal Models, Dalian Medical University, Dalian, Liaoning 116044, China; National Clinical Research Center for Eye Diseases, Shanghai General Hospital, Shanghai Jiao Tong University School of Medicine, Shanghai 200080, China; Institute of Genome Engineered Animal Models for Human Diseases, National Center of Genetically Engineered Animal Models for International Research, Liaoning Province Key Lab of Genome Engineered Animal Models, Dalian Medical University, Dalian, Liaoning 116044, China; Institute of Genome Engineered Animal Models for Human Diseases, National Center of Genetically Engineered Animal Models for International Research, Liaoning Province Key Lab of Genome Engineered Animal Models, Dalian Medical University, Dalian, Liaoning 116044, China; Institute of Genome Engineered Animal Models for Human Diseases, National Center of Genetically Engineered Animal Models for International Research, Liaoning Province Key Lab of Genome Engineered Animal Models, Dalian Medical University, Dalian, Liaoning 116044, China; Jinhua Academy of Zhejiang Chinese Medical University, Jinhua, Zhejiang 310053, China; Institute of Genome Engineered Animal Models for Human Diseases, National Center of Genetically Engineered Animal Models for International Research, Liaoning Province Key Lab of Genome Engineered Animal Models, Dalian Medical University, Dalian, Liaoning 116044, China; Key Laboratory of Endocrine Glucose & Lipids Metabolism and Brain Aging, Ministry of Education, Shandong Provincial Hospital, Science and Technology Innovation Center, Shandong First Medical University & Shandong Academy of Medical Sciences, Jinan, Shandong 250021, China; National Clinical Research Center for Eye Diseases, Shanghai General Hospital, Shanghai Jiao Tong University School of Medicine, Shanghai 200080, China; Center for Life Sciences, School of Life Sciences, Yunnan University, Kunming, Yunnan 650091, China; Institute of Genome Engineered Animal Models for Human Diseases, National Center of Genetically Engineered Animal Models for International Research, Liaoning Province Key Lab of Genome Engineered Animal Models, Dalian Medical University, Dalian, Liaoning 116044, China; Key Laboratory of Endocrine Glucose & Lipids Metabolism and Brain Aging, Ministry of Education, Shandong Provincial Hospital, Science and Technology Innovation Center, Shandong First Medical University & Shandong Academy of Medical Sciences, Jinan, Shandong 250021, China

**Keywords:** MAFLD, growth hormone receptor, liver GHRKO, lean body mass

## Abstract

Metabolic dysfunction-associated fatty liver disease (MAFLD) has become the most prevalent chronic liver disease worldwide, affecting both obese and non-obese individuals. While the reduced levels of circulating growth hormone (GH) and insulin-like growth factor 1 (IGF-1) have been consistently observed in patients with hepatic steatosis, the molecular role of hepatic growth hormone receptor (GHR) in MAFLD pathogenesis remains unclear. In this study, we established a liver-specific *Ghr* knockout (LGHRKO) mouse model that faithfully recapitulates non-obese MAFLD, characterized by hepatomegaly, elevated serum lipids and transaminases, and pronounced hepatic lipid accumulation, all occurring in the absence of obesity or increased adiposity. Mechanistically, LGHRKO livers displayed enhanced lipogenesis, impaired lipolysis, and upregulated cluster of differentiation 36 (CD36) expression, thereby driving hepatic lipid deposition. Single-cell RNA sequencing (scRNA-seq) further revealed hepatocyte-specific transcriptional alterations, including activation of lipid metabolic pathways and dysregulation of autophagy-related processes, providing insights into the cellular mechanisms underlying disease progression. Complementary human genetic analyses, including two-sample Mendelian randomization (MR) and genome-wide association studies (GWASs), demonstrated a causal relationship between impaired GH–IGF signaling and susceptibility to MAFLD, thereby bridging experimental observations with human disease risk. Collectively, our findings identify hepatic GHR deficiency as a key driver of non-obese MAFLD, establish LGHRKO mice as a valuable model for mechanistic and therapeutic studies, and underscore the translational significance of GH–IGF signaling in metabolic liver disease.

## Introduction

Parallel to the global epidemic of metabolic disorders, metabolic dysfunction-associated fatty liver disease (MAFLD) has emerged as the most common chronic liver disease worldwide and is expected to become the leading cause of end-stage liver diseases in the near future [1, 2]. MAFLD encompasses a spectrum of progressive conditions, ranging from simple liver steatosis (nonalcoholic fatty liver [NAFL], characterized by lipid accumulation) to more severe stages such as metabolic dysfunction-associated steatohepatitis (MASH, which involves persistent inflammatory infiltrates or fibrosis), ultimately progressing to cirrhosis and hepatocellular carcinoma (HCC) [3]. The global prevalence of MAFLD is currently estimated at 25%, showing an alarming upward trend [4]. Among those affected, 20% progress to MASH, which has become one of the leading indications for liver transplantation.

Though MAFLD is often diagnosed in obese or at least overweight individuals, it also occurs in lean or normal-weight patients, referred to as lean MAFLD. These individuals are sometimes called metabolically obese normal weight (MONW). The global prevalence of lean MAFLD is estimated to range from 12% to 20% [5]. Unlike the traditional understanding of MAFLD, lean MAFLD shows a higher prevalence in Asia compared to European populations [6]. Compared to obese MAFLD patients, lean MAFLD patients exhibit less insulin resistance but have similar levels of dyslipidemia and a comparable risk of progressing to MASH [7]. Additionally, lean MAFLD patients are at a higher risk for cardiovascular mortality and severe liver disease than their obese counterparts [5, 8]. Studies have suggested that genetic variants in patatin-like phospholipase domain-containing 3 (*PNPLA3*) or transmembrane 6 superfamily member 2 (*TM6SF2*) are associated with lean MAFLD [9]. Nevertheless, the molecular mechanisms underlying lean MAFLD are still unclear.

Growth hormone (GH) regulates somatotropic growth and a variety of physiological processes, such as lipolysis in the liver and adipose tissues [10–12]. The circulating insulin-like growth factor 1 (IGF-1) is primarily secreted by hepatocytes in response to GH stimulation. The prevalence of MAFLD is higher in patients with GH deficiency, and GH supplementation therapy has been shown to improve the histology of MAFLD [13–15]. Patients with dysfunctional growth hormone receptor (GHR), such as those with Laron syndrome [16], are also prone to developing MAFLD [17, 18]. Mice with hepatic GHR deletion or downstream signaling deficiencies exhibit hepatic steatosis, with body weight similar to or lower than that of control mice. Surprisingly, few studies have linked this phenomenon to lean MAFLD [19–21].

In this study, we demonstrated that liver-specific knockout of *Ghr* (LGHRKO) mice showed a phenotype comparable to diet-induced obese (DIO) mice in terms of blood lipid profiles, insulin resistance, and hepatic lipid content. However, these mice exhibited similar body weight and adipose tissue weight as control mice, suggesting that LGHRKO mice could serve as a model to simulate lean MAFLD patients. We observed that the CCAAT/enhancer binding protein (C/EBP) beta (C/EBPβ)-cluster of differentiation 36 (CD36) axis was activated in LGHRKO hepatocytes, promoting hepatic lipid accumulation. Furthermore, we explored the causal relationship between GH levels and nonalcoholic fatty liver disease (NAFLD) by applying two-sample Mendelian randomization (MR) analysis using genome-wide association study (GWAS) catalog summary data. Our findings provide novel evidence that liver-specific GHR ablation accelerates lipid intake and *de novo* lipogenesis while enhancing lipolysis in adipose tissue, leading to the development of lean MAFLD.

## Results

### LGHRKO mice display normal body weight

**Figure 1:**
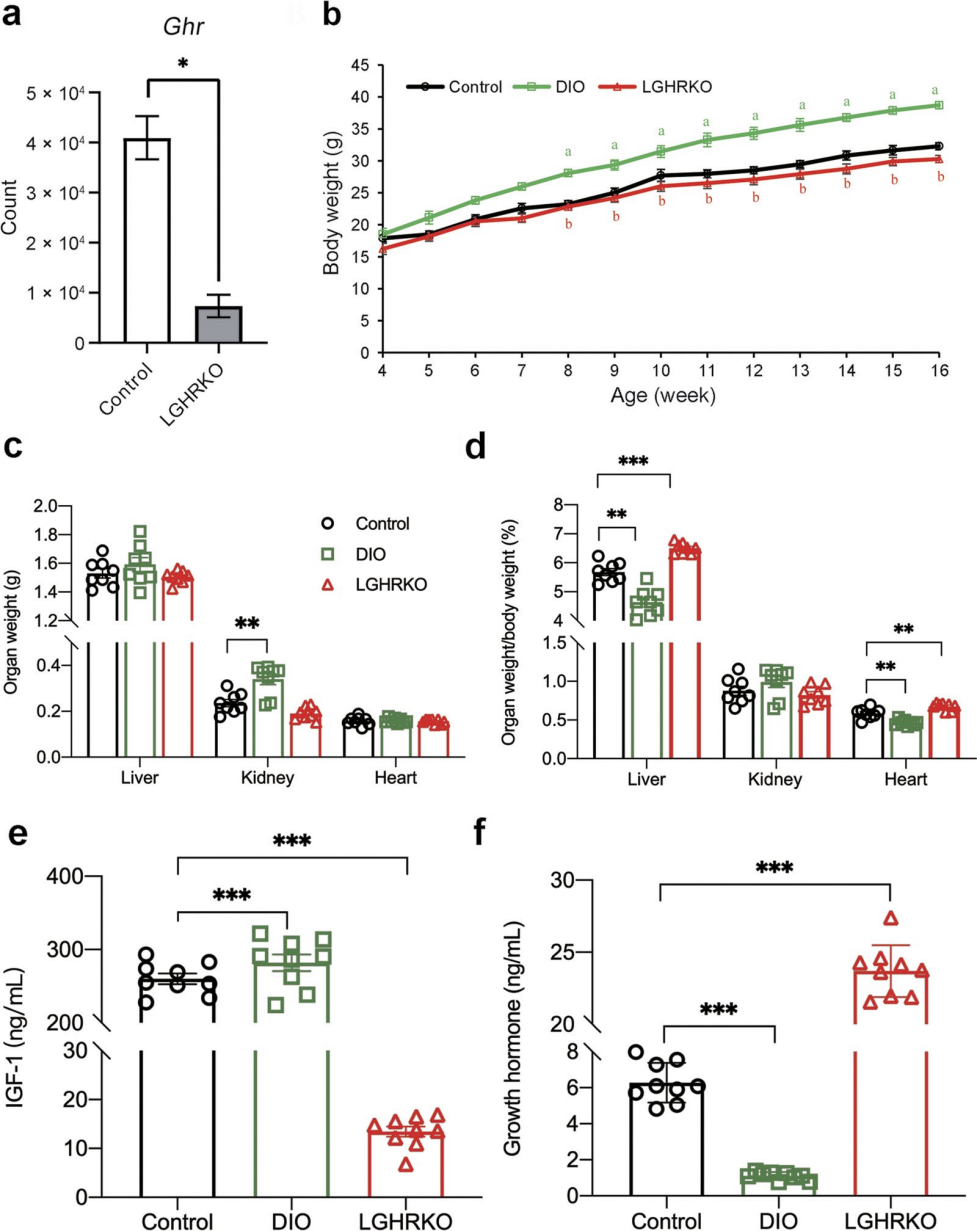
LGHRKO mice display higher liver index and leaner body than DIO mice. (a) The expression level of *Ghr* in the livers of LGHRKO mice. (b) Body weight of control, DIO, and LGHRKO mice followed to 16 weeks. (c) Weights of liver, kidney, and heart in control, DIO and LGHRKO mice. (d) Relative organ weights of liver, kidney, and heart for control, DIO, and LGHRKO mice (organ weight/body weight). (e) Circulating IGF-1 levels in control, DIO, and LGHRKO mice. (f) Circulating GH levels in control, DIO, and LGHRKO mice. *n *≥ 8 mice in each group. Data are presented as mean ± SEM.

Liver-specific GHR deletion in LGHRKO mice was created by crossing GHR LL mice, which contain two LoxP sites flanking exon 4 of the *Ghr* gene, with the *Alb-Cre* mice, where the *Cre* gene was driven by the albumin promoter. Removal of exon 4 of the *Ghr* gene by the Cre enzyme in LGHRKO mice is depicted in Supplementary Fig. S1a. Reduced expression of *Ghr* in the livers of LGHRKO mice was validated by quantifying single-cell sequencing reads (Fig. 1a). Additionally, the expression of *Igf1*, a downstream target of the GHR signaling pathway, was significantly decreased as a result of GHR loss in LGHRKO livers (Supplementary Fig. S1b). As shown in Fig. 1b, LGHRKO mice exhibited similar body weight to control mice from 4 to 16 weeks of age. The body weight of LGHRKO mice was significantly lower than that of DIO mice starting at 8 weeks, with the weight gap between the two groups widening as the mice aged (Fig. 1b). In terms of major organs, the weights of the liver and heart showed no difference between LGHRKO, DIO, and control mice. Only kidney weight was significantly increased in DIO mice (Fig. 1c). Upon calculating the relative organ weight, we found that LGHRKO mice had the highest relative liver weight. The relative heart weight in control mice was also significantly higher than that in DIO mice, with no difference observed in the relative kidney weight among the three groups (Fig. 1d). Given that body growth is primarily regu­lated by GH and IGF-1, and the modifications to LGHRKO mice might affect the levels of these hormones, we examined the serum levels of GH and IGF-1 across the three groups of mice. The IGF-1 levels in LGHRKO mice were significantly lower than those in control and DIO mice (Fig. 1e; Supplementary Fig. S1b). However, the GH levels in LGHRKO mice were significantly higher than those in control mice, and the GH levels were further decreased in DIO mice (Fig. 1f). Taken together, these results indicate that LGHRKO mice have increased relative liver weight, normal body weight, decreased serum IGF-1 content, and increased serum GH content.

### LGHRKO mice exhibit hyperlipidemia similar to the state of DIO mice

**Figure 2:**
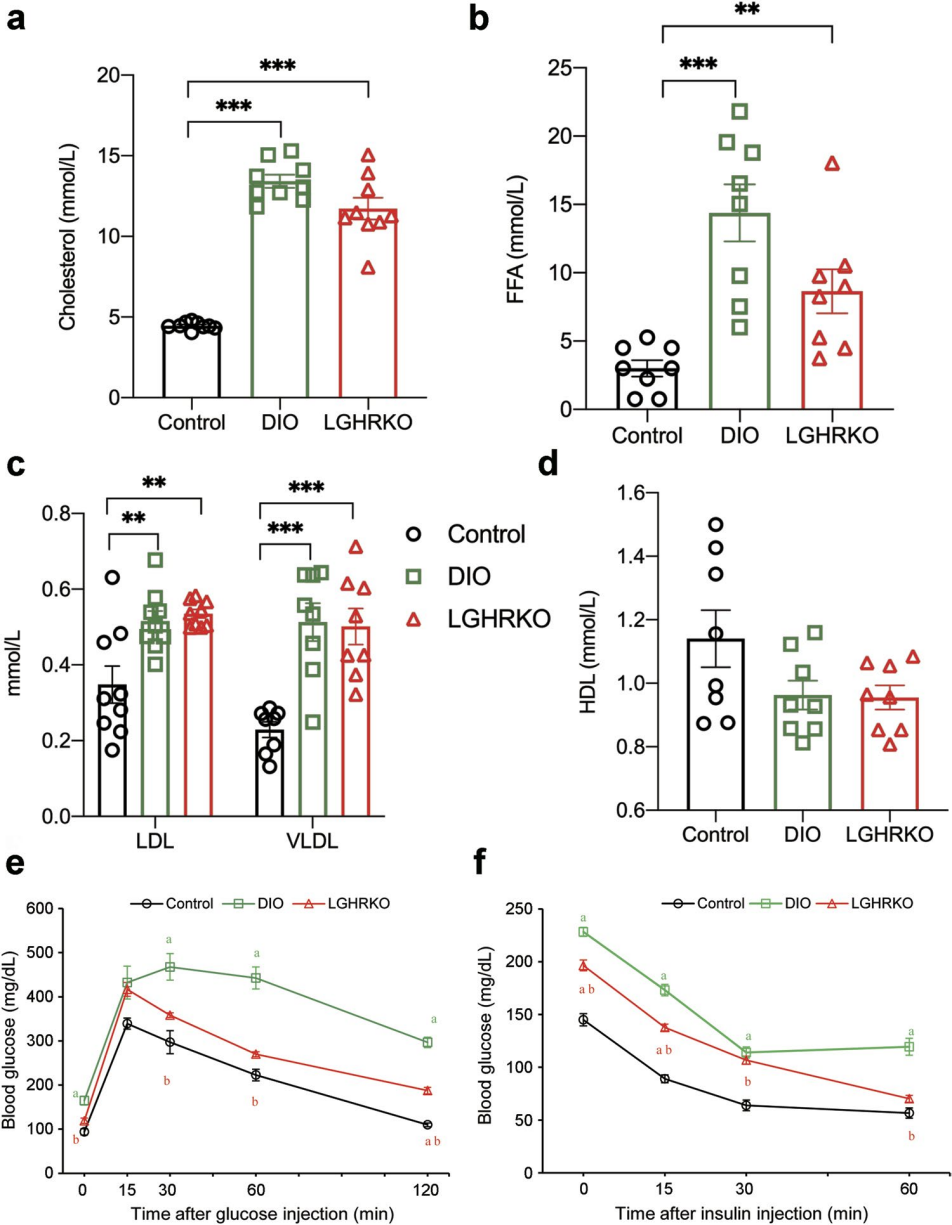
LGHRKO mice display similar serum lipid profiles and glucose metabolism as DIO mice. (a) Serum cholesterol levels in control, DIO, and LGHRKO mice. (b) Serum FFA levels in control, DIO, and LGHRKO mice. (c) Serum LDL and VLDL levels in control, DIO, and LGHRKO mice. (d) Serum HDL levels in control, DIO, and LGHRKO mice. (e) GTTs of control, DIO, and LGHRKO mice. (f) ITTs of control, DIO, and LGHRKO mice. *n *= 6–9 mice in each group. Data are presented as mean ± SEM.

Both lean MAFLD and obese MAFLD are characterized by hyperlipi­demia [7]. Additionally, a key factor in determining whether LGHRKO mice can serve as a lean MAFLD animal model is whether their blood lipid content is comparable to that of DIO mice. The liver plays a crucial role in lipid homeostasis, and hepatic GHR deficiency has previously been shown to increase the levels of plasma very low-density lipoprotein (VLDL), low-density lipoprotein (LDL), and cholesterol [22]. Elevated serum cholesterol levels are statistically associated with fatty liver disease [23]. We found that serum cholesterol content was significantly elevated in both LGHRKO and DIO mice [24–26] (Fig. 2a).

Clinically, high serum free fatty acids (FFAs) are associated with steatosis [27], as shown in both obese MAFLD [28] and lean MAFLD [29]. The FFA content in LGHRKO mice was significantly higher than that in control mice, although it was lower than that in DIO mice (Fig. 2b). Additionally, circulating LDL are considered indicators of fatty liver [30], and MAFLD is often associated with increased blood levels of VLDL [31]. In our study, serum LDL and VLDL levels were significantly increased in both LGHRKO and DIO mice, with no apparent difference observed between the two groups (Fig. 2c). High-density lipoprotein (HDL) is another biomarker for steatosis [32], and it is also reduced in patients with lean MAFLD [6]. Both LGHRKO and DIO mice exhibited lower HDL levels, although this difference was not statistically significant (Fig. 2d). Taken together, these data suggest that LGHRKO mice display hyperlipidemia ­similar to that observed in DIO mice, despite having relatively lean body weight. This further supports the notion that LGHRKO mice may serve as a model for studying lean MAFLD.

### LGHRKO mice exhibit imbalanced glucose tolerance and insulin resistance

Insulin resistance is a crucial factor in the initiation and progression of MAFLD. Clinical studies have also shown that lean MAFLD patients exhibit impaired glucose tolerance when compared to lean individuals without MAFLD [5, 33]. To assess these features in LGHRKO mice, we ran a glucose tolerance test (GTT). The blood glucose levels of LGHRKO mice were higher than those of control mice during the test, but lower than those of DIO mice from 30 min to 120 min after glucose injection (Fig. 2e). When we calculated the area under the curve (AUC), the GTT AUC of LGHRKO mice was significantly higher than that of control mice but still lower than that of DIO mice (Supplementary Fig. S2a). This result indicates that LGHRKO mice have impaired glucose tolerance, despite not being obese.

Insulin resistance is closely linked to MAFLD, with the most severe insulin resistance observed in obese MAFLD patients, followed by lean MAFLD, obese individuals without MAFLD, and lean individuals without MAFLD [33]. In this study, we evaluated insulin resistance, which was measured by an insulin tolerance test (ITT). The blood glucose levels of LGHRKO mice were significantly higher than those of control mice but lower than those of DIO mice at most time points during the ITT (Fig. 2f). The AUC calculation of ITT further confirmed that LGHRKO mice had higher insulin resistance than control mice, though it was still less than that observed in DIO mice (Supplementary Fig. S2b). These findings highlight that LGHRKO mice exhibit impaired glucose tolerance and insulin resistance, characteristics that are commonly observed in lean MAFLD patients, despite the absence of obesity.

To test whether insulin sensitivity was impaired in the livers of GHR KO mice, we first performed gene set enrichment analysis (GSEA) using a curated list of insulin signaling pathway genes, including insulin receptor substrate 1 (*Irs1*), *Irs2*, phosphatidylinositol-4,5-bisphosphate 3-kinase catalytic subunit alpha (*Pik3ca*), *Pik3cb*, *Pik3cd*, *Pik3cg*, AKT serine/threonine kinase 1 (*Akt1*), *Akt2*, *Akt3*, mitogen-activated protein kinase 1 (*Mapk1*), *Mapk3*, glycogen ­synthase kinase 3 beta (*Gsk3b*), Forkhead box O1 (*Foxo1*), sterol ­regulatory element binding transcription factor 1 (*Srebf1*), glucokinase (*Gck*), solute carrier family 2 member 4 (*Slc2a4*), hexokinase 2 (*Hk2*), phosphofructokinase muscle-type (*Pfkm*), protein kinase AMP-activated catalytic subunit α 1 (*Prkaa1*), *Prkaa2*, mechanistic target of rapamycin (*Mtor*), kinase ribosomal protein S6 kinase beta-1 (*Rps6kb1*), and eukaryotic translation initiation factor 4E binding protein 1 (*Eif4ebp1*). No significantly changes in these gene sets were observed at the transcriptomic level (Supplementary  Fig. S2c). However, since the insulin signaling ­pathway is primarily regulated through post-translational modifications, particularly protein phosphorylation, transcriptomic data alone may not fully reflect functional alterations. Therefore, we next assessed the phosphorylation level of key signaling proteins, including protein kinase B (Akt), phosphoinositide 3-kinase (PI3K), and mTOR, as well as the expression of sterol regulatory element-binding protein-1c (Srebp-1c) at the protein level. The results showed that total protein levels of Akt, PI3K, and mTOR were not significantly affected by liver-specific GHR deletion (Supplementary Fig. S2d). In contrast, the phosphorylation levels of PI3K, Akt, and mTOR were markedly reduced in GHR KO liver cells, indicating impaired insulin signaling. These findings were further supported by immunohistochemistry analysis of liver tissues, which confirmed decreased levels of p-AKT, p-PI3K, and p-mTOR in GHR KO mice (Supplementary Fig. S2e).

### LGHRKO mice show hepatic steatosis and liver dysfunction

**Figure 3:**
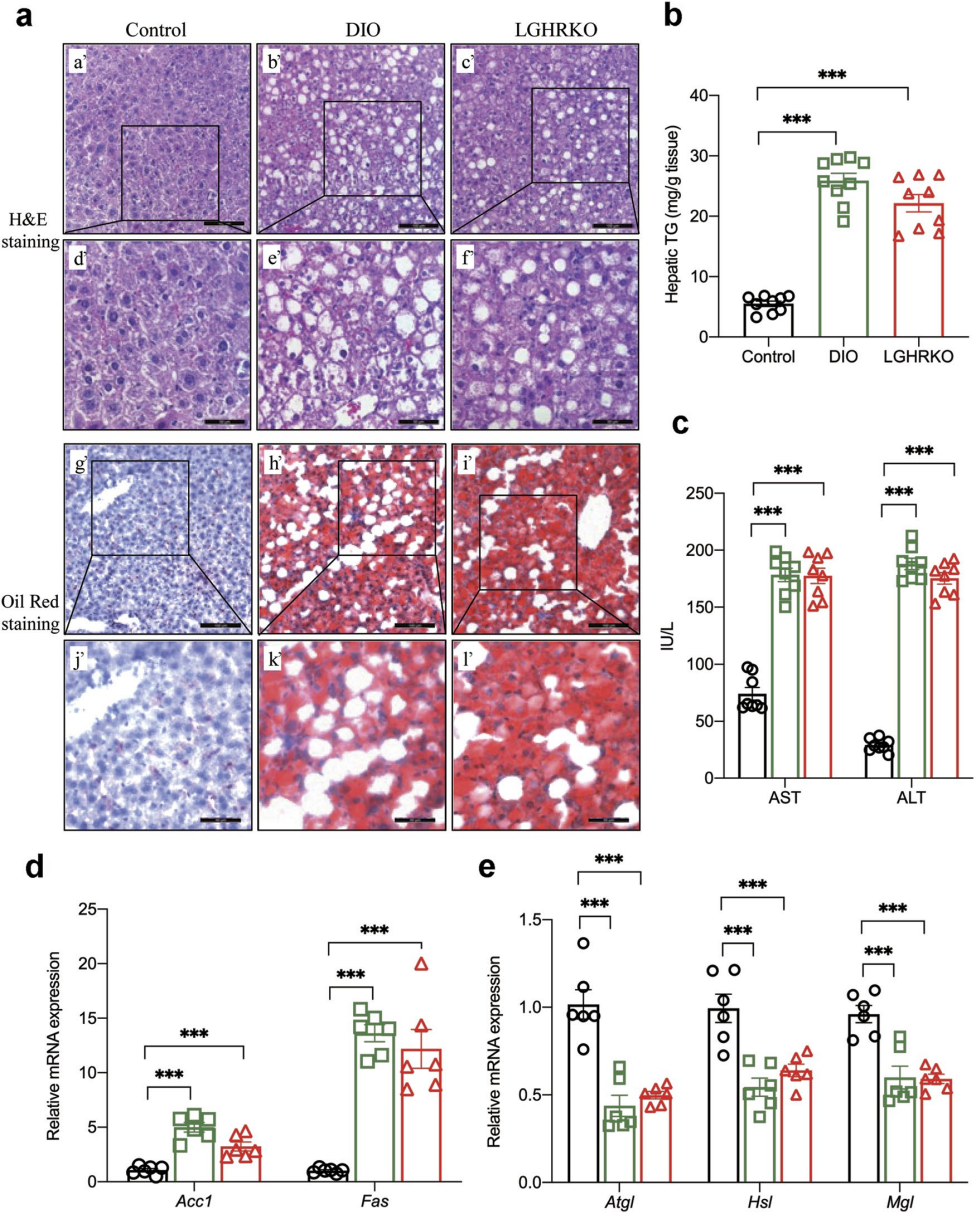
LGHRKO mice contain excessive hepatic lipids with impaired liver function, similar to DIO mice. (a) Microscopic appearance of the livers in control, DIO, and LGHRKO mice. (a’–c’) H&E-stained liver section at 200× magnification. (d’–f’) H&E-stained liver section at 400× magnification. (g’–i’) Oil Red O-stained liver section at 200× magnification. (j’–l’) Oil Red O-stained liver section at 400× magnification. (b) Hepatic TG content of control, DIO, and LGHRKO mice. (c) Serum AST and ALT levels of control, DIO, and LGHRKO mice. (d) Relative hepatic mRNA levels of lipogenesis genes *Acc1* and *Fas*. (e) Relative hepatic mRNA levels of lipolysis genes *Atgl*, *Hsl*, and *Mgl*. *n *= 6–8 mice in each group. Data are presented as mean ± SEM.

A meta-analysis has reported that lean MAFLD tends to show less severe hepatic histology compared to obese MAFLD [34]. In this study, histological staining showed hepatocyte ballooning in both DIO and LGHRKO mice (Fig. 3a a’−f’). Although the number of ballooning cells was slightly lower in LGHRKO mice than that in DIO mice, it was significantly higher than that in control mice. Oil Red O staining, which highlights lipid droplets in red, demonstrated abundant red staining in both LGHRKO and DIO mouse livers, indicating substantial lipid accumulation (Fig. 3a g’−l’). The hepatic triglyceride (TG) content in LGHRKO mice was significantly elevated, reaching levels similar to those observed in DIO mice (Fig. 3b). Increased levels of liver enzymes, such as aspartate aminotransferase (AST) and alanine aminotransferase (ALT), are commonly observed in lean MAFLD patients [35]. We found the same pattern in LGHRKO mice. Both AST and ALT levels were significantly elevated in LGHRKO and DIO mice (Fig. 3c). The excessive lipid accumulation in the livers of LGHRKO mice can be attributed to increased expression of lipogenesis genes, such as acetyl-CoA carboxylase 1 (*Acc1*) and fatty acid synthase (*Fas*) (Fig. 3d). In contrast, the expression levels of lipolysis-related genes, including adipose triglyceride lipase (*Atgl*), hormone-sensitive lipase (*Hsl*), and monoacylglycerol lipase (*Mgl*), were reduced in LGHRKO mice (Fig. 3e). Importantly, no significant difference in the expression of these genes was observed between LGHRKO and DIO mice. These results demonstrate that LGHRKO mice exhibit excessive hepatic lipid accumulation, impaired liver function, and an imbalance between lipogenesis and lipolysis in the liver. Notably, the liver histology of LGHRKO mice closely resembles that of DIO mice.

### LGHRKO mice progress to MASH at 16 weeks

**Figure 4:**
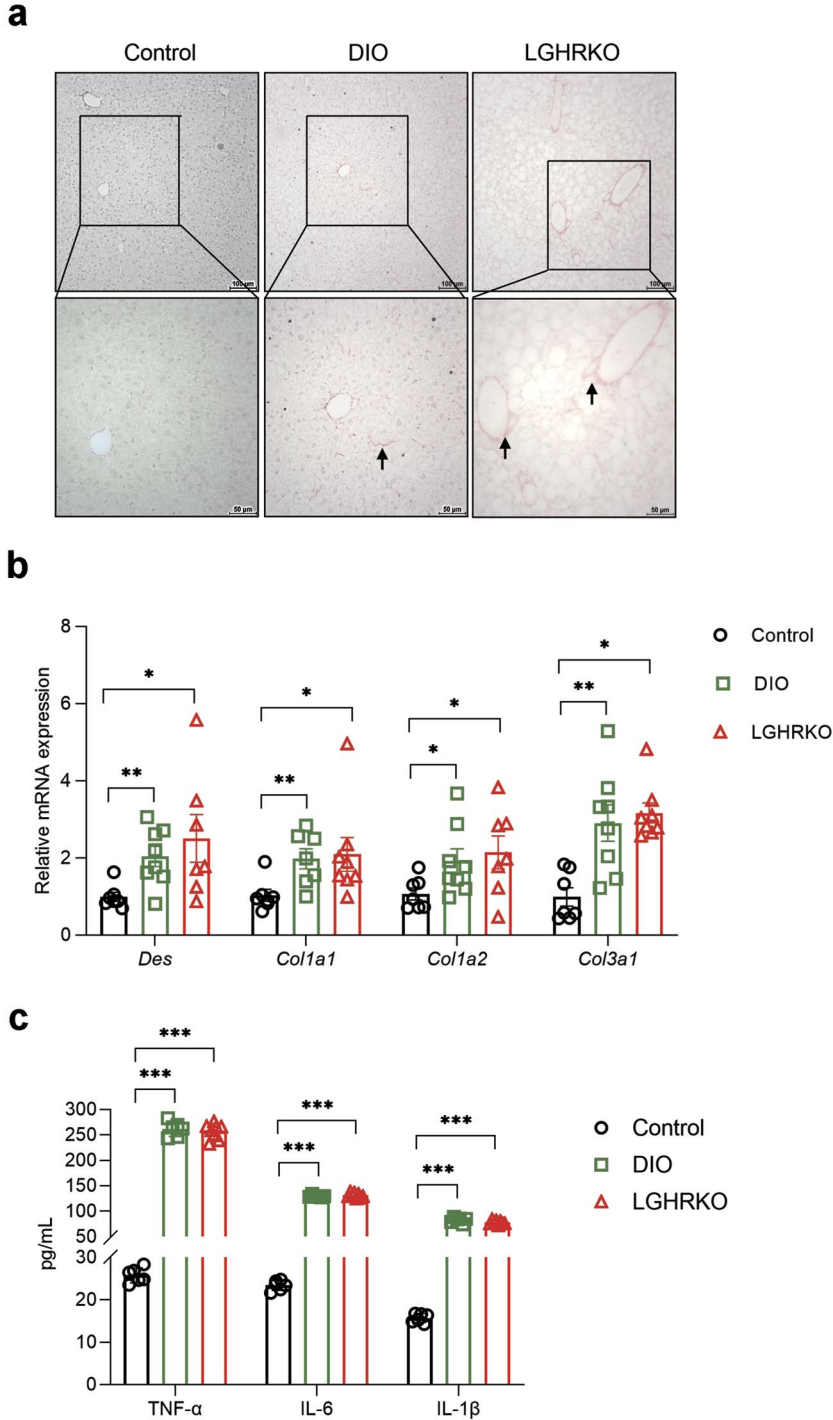
Steatosis in LGHRKO mice deteriorates into MASH at older age. (a) Sirius Red staining showing liver collagen in DIO and LGHRKO mice. Arrows show collagen deposition between hepatocytes (DIO mice) or surrounding vessels (LGHRKO mice). (b) Relative hepatic mRNA levels of fibrogenic genes. (c) The content of inflammatory cytokines. *n *= 6−8 mice in each group. Data are presented as mean ± SEM.

MASH is characterized by lobular inflammation and fibrosis. Liver tissues of 16-week-old mice were stained with Sirius Red to identify fibrosis. Collagen deposition was observed in the livers of LGHRKO and DIO mice (Fig. 4a). In addition, the expression levels of fibrogenesis-related genes, including desmin (*Des*), collagen type 1 alpha 1 (*Col1a1*), *Col1a2*, and *Col3a1*, were significantly elevated in LGHRKO mice (Fig. 4b). Moreover, inflammatory cytokines such as tumor necrosis factor alpha (TNF-α), interleukin (IL)-6, and IL-1β were highly expressed in LGHRKO and DIO mice (Fig. 4c). These findings suggest that steatosis in LGHRKO mice has progressed into MASH.

### LGHRKO mice contain less adipose tissues than DIO mice

**Figure 5:**
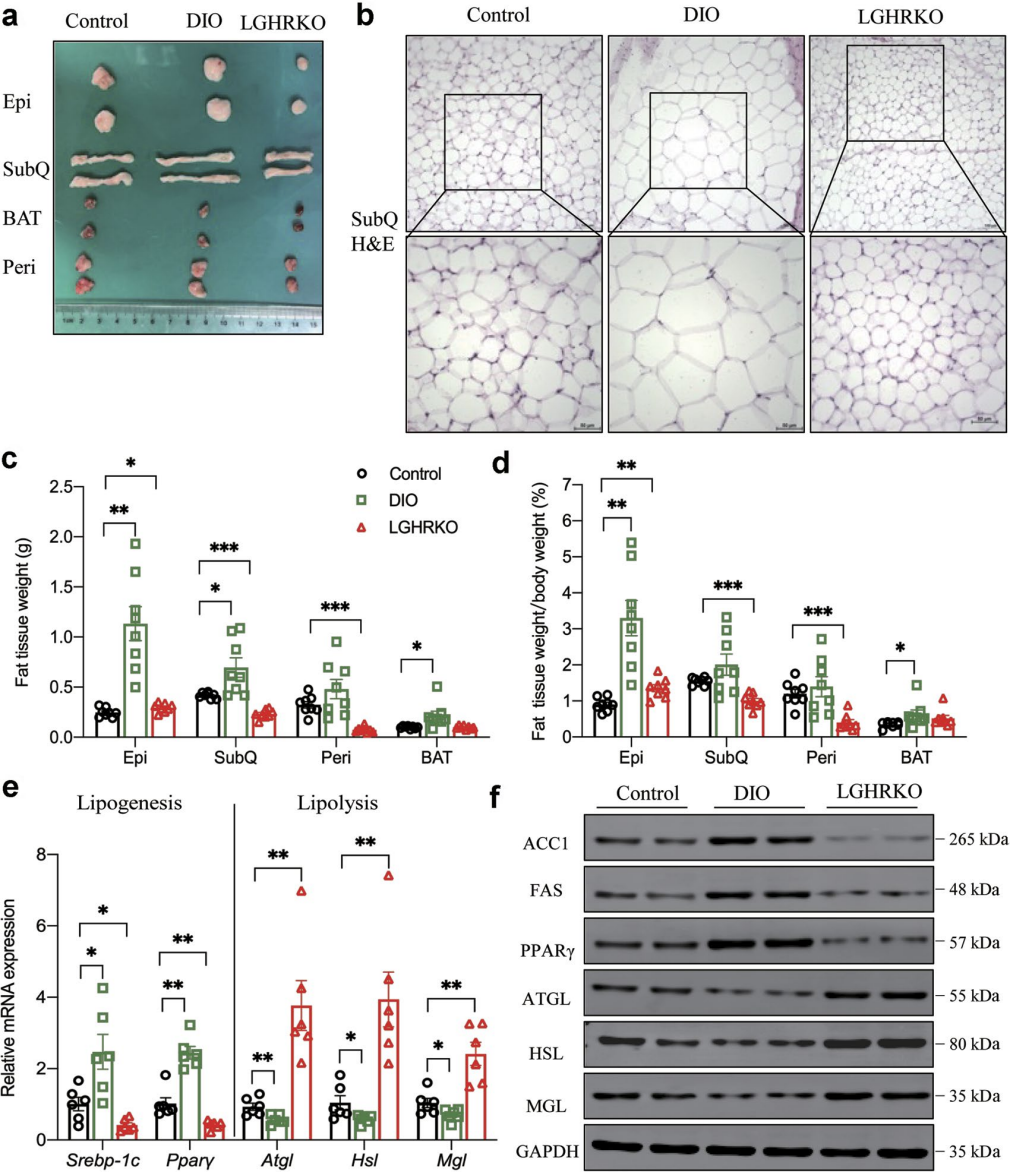
Adipose tissue size is reduced in LGHRKO mice. (a) Normal appearance of fat tissues in control, DIO, and LGHRKO mice. (b) H&E-stained SubQ fat tissues in control, DIO, and LGHRKO mice at 200× or 400× magnification. (c) Weights of different types of fat tissues in control, DIO, and LGHRKO mice. (d) Relative weights of different types of fat tissues in control, DIO, and LGHRKO mice. (e) Relative mRNA levels of lipogenesis- and lipolysis-related genes in the SubQ of control, DIO, and LGHRKO mice. (f) Western blot analysis of lipogenesis- and lipolysis-related proteins in the SubQ of control, DIO, and LGHRKO mice. BAT: brown adipose tissue; Epi: epididymal white adipose tissue; Peri: perirenal white adipose tissue; SubQ: subcutaneous white adipose tissue. *n *= 6 mice in each group. Data are presented as mean ± SEM.

Our previous results showed that LGHRKO mice are not obese based on body weight. To further investigate whether they are lean in terms of fat tissues, adipose tissues from various body parts were excised en bloc and weighed. The gross appearance of adipose tissues collected from LGHRKO, DIO, and control mice revealed that fat tissues in LGHRKO mice were markedly smaller than those in DIO mice, regardless of epididymal fat (Epi), subcutaneous fat (SubQ), brown adipose tissue (BAT), or perirenal fat (Peri) (Fig. 5a). Histological examination showed that SubQ adipocytes in LGHRKO mice were substantially smaller than those in DIO mice and generally smaller than those in control mice (Fig. 5b). The smaller size of adipocytes indicated reduced lipid storage in adipose tissues, contributing to lower adipose tissue weight. By comparing adipose tissue weight across the three groups, we found that Epi, SubQ, BAT, and Peri adipose tissues were all significantly lighter in LGHRKO mice than those in DIO mice. Notably, SubQ and Peri fat in LGHRKO mice even weighed less than those in control mice (Fig. 5c). Regarding relative fat tissue weight, Epi, SubQ, and Peri fat tissues were significantly lighter in LGHRKO than those in DIO mice, with relative Peri fat also being lower in LGHRKO mice than those in control mice (Fig. 5d).

Consistently, mRNA expression levels of lipogenic genes, including *Srebp-1c* and peroxisome proliferator-activated receptor γ (*Pparγ*), were significantly reduced in SubQ adipocytes of LGHRKO mice, followed by control and DIO mice (Fig. 5e). Meanwhile, mRNA expression levels of lipolytic genes such as *Atgl*, *Hsl*, and *Mgl* were dramatically elevated in LGHRKO mice but significantly decreased in DIO mice compared with those in control mice (Fig. 5e). Similarly, protein levels of lipogenic genes (such as ACC1, FAS, and PPARγ) and lipolytic genes (such as ATGL, HSL, and MGL) in SubQ tissues exhibited the same trend in LGHRKO mice (Fig. 5f).

### Single-cell RNA sequencing identifies distinct cell clusters in LGHRKO mice

The *Ghr* knockout in hepatocytes profoundly influenced hepatic metabolism. To identify a deep transcriptional map of the hepatocytes and functionally related non-parenchymal cells, we applied liver single-cell dissociation procedure and single-cell RNA sequencing technique. The two-dimensional (2D) visualization of the cells is presented using t-distributed stochastic neighbor embedding (t-SNE) (Supplementary Fig. S3a). The library size of each cell was counted by unique molecular identifier with consi­derable variation among cells, indicating potential differences in biological activities (Supplementary Fig. S3a). A total of 66,493 cells were clustered into 21 populations (Supplementary Fig. S3b). Each cluster was composed of liver cells from both control and LGHRKO mice (Supplementary Fig. 3c). The identified markers of each cluster are shown in Supplementary Fig. S4a. However, the contribution of mice in each group to the cluster differed. Clusters 6 and 7 mainly consisted of control samples, whereas Clusters 8, 11, 12, 16, 17, and 18 predominantly comprised LGHRKO samples. The remaining clusters contained almost equal proportions of control and LGHRKO samples (Supplementary Fig. S3b and c). Clusters 0–10, 13–15, and 18 were identified as hepatocytes, marked by highly expressed cytochrome P450 (CYP) gene family, major urinary protein (MUP) gene family, sulfotransferase (SULT) gene family, argininosuccinate synthetase 1 (*Ass1*), glutamate-ammonia ligase (*Glul*), histidine ammonia lyase (*Hal*), ornithine aminotransferase (*Oat*), insulin-like growth factor-binding protein-1 (*Igfbp1*), stearoyl-CoA desaturase-1 (*Scd1*), hydroxysteroid 17-beta dehydrogenase 13 (*Hsd17b13*), and ferritin heavy chain 1 (*Fth1*), among others. Clusters 11, 16, and 17 were macrophages, marked by highly expressed chemokine C-C motif ligands 4 (*Ccl4*), *Ccl5*), whey acidic protein four-disulfide core domain 17 (*Wfdc17*), thymosin beta 4 X-linked (*Tmsb4x*), Fc epsilon receptor I gamma chain (*Fecr1g*), interferon alpha-inducible protein 27 like 2 A (*Ifi27l2a*), and *Cd52.* Cluster 12 represented endothelial cells, marked by highly expressed secreted protein acidic and rich in cysteine (*Sparc*), *Igfbp7*, protein tyrosine phosphatase receptor type B (*Ptprb*), and transmembrane-4 L-six family member-1 (*Tm4sf1*). Cluster 19 was composed of T cells and natural killer (NK) cells, marked by highly expressed thymosin beta10 (*Tmsb10*), cysteine-rich protein 1 (*Crip1*), *Ccl5*, and *Il4*. Cluster 20 comprised endothelial cells, fibroblasts, and hepatocytes, marked by highly expressed decorin (*Dcn*), extracellular matrix protein 1 (*Ecm1*), biglycan (*Bgn*), and regulator of G-protein signaling 5 (*Rgs5*). The exact cell type composition of each cluster is listed in Table 1.

**Table 1:** Cell type composition of each cluster.

Cell type Cluster	0	1	2	3	4	5	6	7	8	9	10	11	12	13	14	15	16	17	18	19	20
B cells	0	0	0	0	0	0	0	0	0	0	0	71	1	0	0	0	0	0	0	2	0
Dendritic cells	0	0	0	0	0	0	0	0	0	0	0	2	0	0	0	0	0	1	0	3	0
Endothelial cells	0	0	0	0	0	0	0	0	0	0	0	0	1815	0	0	0	5	9	0	2	30
Erythrocytes	0	0	0	0	0	0	0	0	0	0	0	0	0	0	0	0	0	3	0	0	0
Fibroblasts	0	0	0	0	0	0	0	0	0	0	0	0	1	0	0	0	0	2	0	0	26
Granulocytes	0	0	0	0	0	0	0	0	0	0	0	19	0	0	0	0	0	0	0	0	0
Hepatocytes	6781	5526	4985	4864	4829	4191	3999	3322	3019	2991	2549	72	353	1075	820	754	10	332	358	19	17
Macrophages	0	0	5	0	0	0	0	0	0	0	1	2151	3	0	0	0	732	85	0	3	3
Monocytes	0	0	0	0	0	0	0	0	0	0	0	203	3	0	0	0	3	172	0	28	0
NK cells	0	0	0	0	0	0	0	0	0	0	0	5	0	0	0	0	0	0	0	28	0
T cells	0	0	0	0	0	0	0	0	0	0	0	11	0	0	0	0	0	0	0	65	0

### Macrophage cluster enriched in LGHRKO mice is highly related to MAFLD

**Figure 6:**
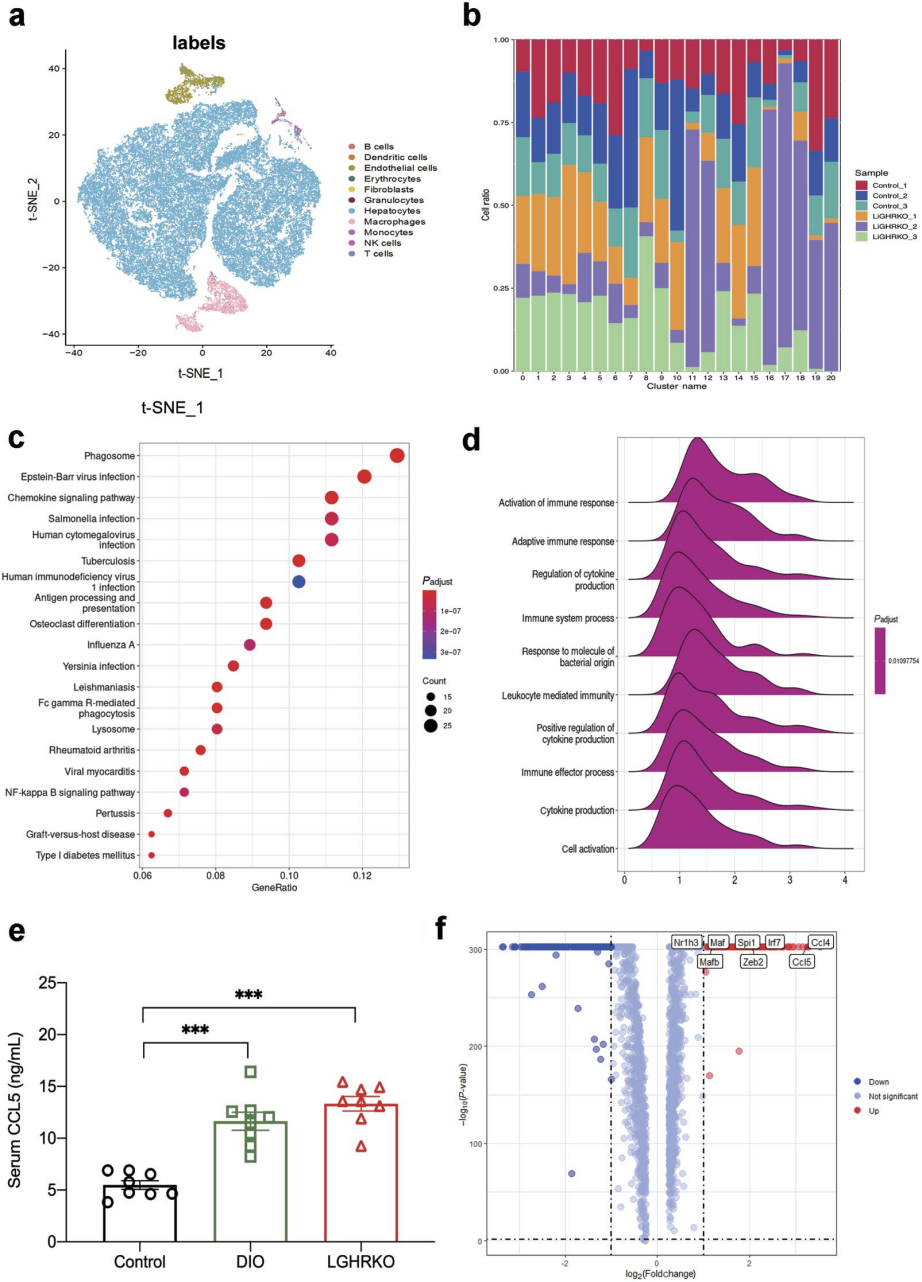
Macrophage cluster enriched in LGHRKO mice is highly related to MAFLD through single-cell sequencing and cell population identification. (a) Cluster map of t-SNE visualized liver cells based on 66,493 single-cell transcriptomes. The cell types of the clusters are noted on the colors and labels. (b) The constitution of clusters from each liver sample. (c) KEGG analysis of Cluster 11. (d) GO analysis of Cluster 11. (e) Serum CCL5 levels of control, DIO, and LGHRKO mice. (f) Volcano plots of up-regulated and down-regulated genes in Cluster 11.

Each cell in the single-cell RNA sequencing was annotated into different cell type categories using the SingleR package and Mouse RNA-seq Data (Fig. 6a), including B cells, dendritic cells, endothelial cells, erythrocytes, fibroblasts, granulocytes, hepatocytes, macrophages, monocytes, NK cells, and T cells. The exact cell type composition of each cluster is listed in Table 1. Among the clusters with a relatively large population, Cluster 11 was mostly composed of LGHRKO cells (Fig. 6b), which were macrophage cells. Apart from hepatocytes, macrophage cells are a major contributing factor in the development of steatosis [36]. Therefore, we further investigated this cluster. Kyoto Encyclopedia of Genes and Genomes (KEGG) analysis of upregulated genes in Cluster 11 revealed its significant association with the chemokine signaling pathway (Fig. 6c), suggesting that this cluster was heavily involved in cytokine signaling. Gene Ontology (GO) analysis showed that the upregulated genes in Cluster 11 were significantly enriched in the regulation of cytokine production (Fig. 6d). The feature genes of Cluster 11 included *Ccl4*, *Wfdc17*, *Ccl5*, and *Fcer1g* (Supplementary Fig. S4b). Both *Ccl4* and *Ccl5* are cysteine-cysteine (CC) family chemokine members and proinflammatory cytokines secreted by monocytes [37]. MAFLD patients exhibit upregulated hepatic and blood CCL5 levels [38], and an *in vivo* study has demonstrated that CCL5 contributes to fibrosis in MAFLD [39].

We next measured the serum levels of CCL5 in control, DIO, and LGHRKO mice and found that CCL5 levels were elevated in both LGHRKO and DIO mice (Fig. 6e). Volcano plots showed that nuclear receptor subfamily 1 group H member 3 (*Nrlh3*)*,* MAF bZIP transcription factor B (*Mafb*)*, Maf*, Spi-1 proto-oncogene (*Spi1*)*,* zinc finger E box-binding homeobox 2 (*Zeb2*), interferon regulatory factor 7 (*Irf7*), *Ccl4*, and *Ccl5* were elevated in Cluster 11 (Fig. 6f). IRF7, a member of the IRF family, binds to myeloid differentiation primary response 88 (MyD88) to form a complex to initiate downstream gene expression [40]. It is upregulated in obese mice and humans, and knockout of IRF7 protects mice from DIO-induced insulin resistance and obesity [41]. IRF7 is also one of the key activators of *Ccl5* [43]. Furthermore, PU.1 (encoded by *Spi1*) is elevated in DIO-induced obese mice, and knockdown of this gene in macrophages attenuates hepatic liver MASH in *db*/*db* mice [42]. PU.1 has also been reported to interact with IRF8 to activate *Ccl5* transcription [44]. Additionally, a few fibroblasts were identified in Cluster 20, which consisted of endothelial cells, fibroblasts, and hepatocytes. This suggests that macrophage-mediated inflammatory responses may induce fibrogenesis during liver steatosis and/or MASH.

### Hepatocyte cluster enriched in LGHRKO mice has high expression of C/EBPβ and CD36

Hepatocytes are the main component of the liver and play a pivotal role in lipid metabolism. Clusters 8 and 18 were both hepatocytes mainly composed of LGHRKO samples, with Cluster 8 having a relatively larger population than Cluster 18 (Fig. 6b). Therefore, we focused on the expression characteristic of Cluster 8. By separating cells contributed by control or LGHRKO mice in Cluster 8 in t-SNE visualization, we counted 888 cells from control mice and 2131 cells from LGHRKO mice (Supplementary Fig. S3d). This cluster was enriched for the expression of dihydropyrimidinase (*Dpys*), mitochondrially encoded NADH dehydrogenase 3 (*mt-Nd3*), cytochrome P450 family 2 subfamily B member 13 (*Cyp2b13*), and *Cebpb* ­(Supplementary  Fig. S4c). High expression of *mt-Nd3* has been reported to be accompanied by histological severity of steatosis [45], which aligns with the situation in this cluster.

**Figure 7:**
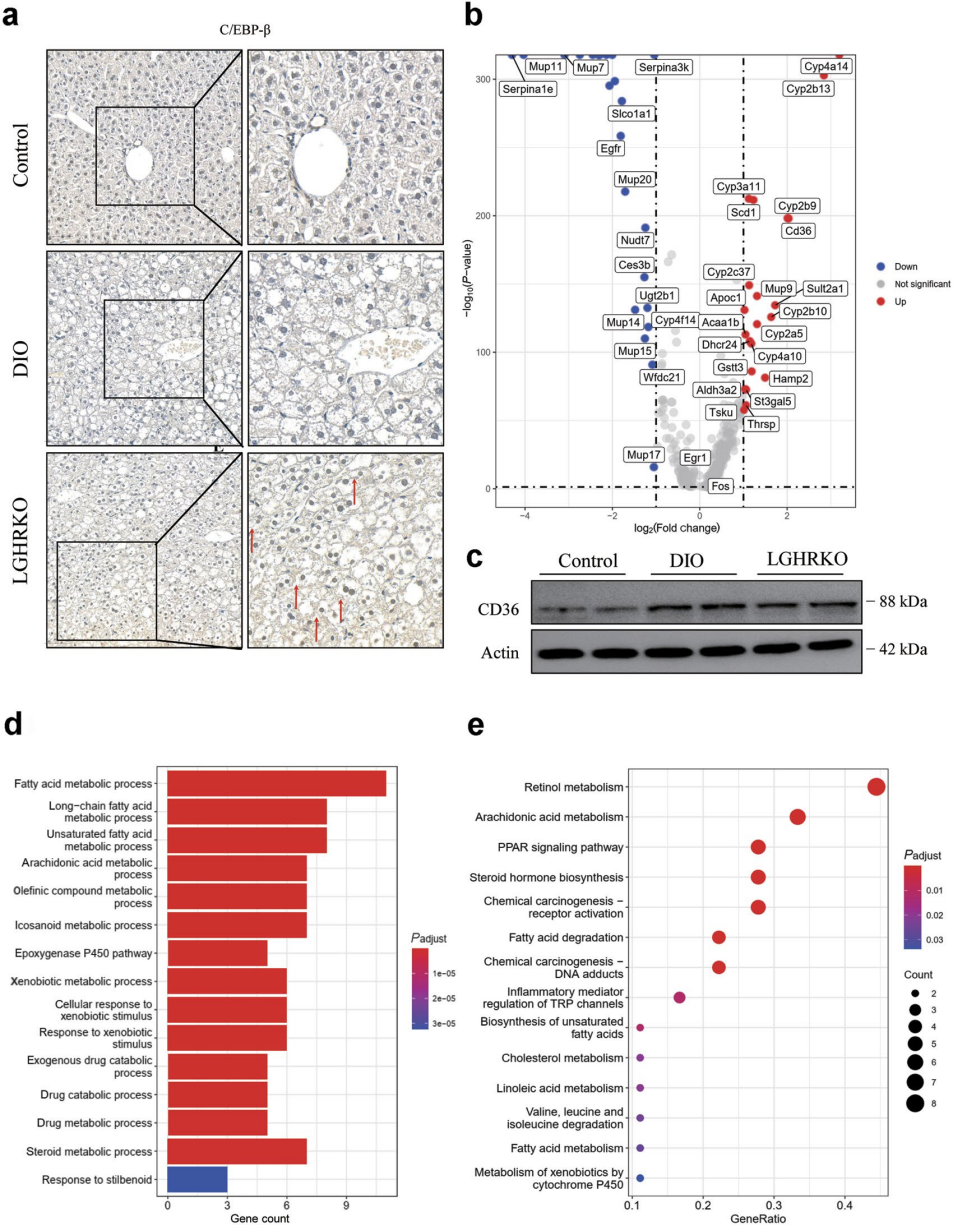
Hepatocyte cluster enriched in LGHRKO mice displays high expression of C/EBPβ and CD36. (a) Liver sections from control, DIO, and LGHRKO mice immunostained with anti-C/EBPβ antibody. Red arrow pointing to anti-C/EBPβ-positive nucleus. (b) Volcano plots of up-regulated and down-regulated genes in Cluster 8. (c) Western blot analysis revealing increased CD36 content in LGHRKO and DIO mice. (d) GO analysis of upregulated pathways in Cluster 8. (e) KEGG analysis of upregulated pathways in Cluster 8.

C/EBPβ (protein encoded by *Cebpb*) regulates hepatic steatosis, inflammation, and endoplasmic reticulum (ER) stress. Deficiency of this gene attenuates lipid accumulation and inflammation in MASH [46]. The liver section of LGHRKO mice showed significantly more C/EBPβ than those of control mice (red arrows), whereas less positive staining was observed in the nuclei of DIO mice (Fig. 7a). The expression level of *Cebpb* was not significantly altered in the livers of LGHRKO mice (Supplementary Fig. 5a), indicating that the regulatory function of C/EBPβ on its downstream target genes may be governed primarily at the protein level rather than by changes in transcription. This observation indicates that C/EBPβ might be one of the driving forces of lipid deposition in the livers of LGHRKO mice. Apart from lipogenesis genes, C/EBPβ also mediates *Cd36* transcription [47, 48]. Genes such as *Cyp2b13*, *Cyp4a14*, *Cyp2b29*, and *Cd36* were highly expressed in LGHRKO cells in Cluster 8, while genes such as *Mup7*, *Mup11*, solute carrier organic anion transporter family, member 1a1 (*Slco1a1*), and epidermal growth factor receptor (*Egfr*) exhibited low expression in LGHRKO cells of Cluster 8 (Fig. 7b).

CD36 is the main transporter of FFA, carrying extracellular FFA into hepatocytes. As a downstream target gene of C/EBPβ, *Cd36* expression was elevated in the livers of LGHRKO mice ­(Supplementary  Fig. S5b) and specifically in hepatocytes (Fig. 7b). This upregulation was consistent with the increased nuclear localization of C/EBPβ observed in LGHRKO mice (Fig. 7a). Western blot analysis showed that the protein levels of CD36 were elevated in LGHRKO and DIO mice (Fig. 7c), suggesting increased FFA uptake in the liver. The featured genes and upregulated transcription factors were both related to the progression of MAFLD. Our findings indicate that the upregulated C/EBPβ and CD36 might be responsible for the unbalanced lipid metabolism in hepatocytes of LGHRKO mice. Differentially expressed genes (DEGs) in the fatty acid metabolic process, long-chain fatty acid metabolic process, and unsaturated fatty acid metabolic process were significantly activated in GO analysis of Cluster 8 (Fig. 7d). Similarly, KEGG analysis of upregulated genes in Cluster 8 found that pathways related to arachidonic acid metabolism, fatty acid degradation, and cholesterol metabolism were activated (Fig. 7e). Meanwhile, the biosynthesis pathway of unsaturated fatty acids was also enriched. The findings from GO and KEGG analyses implied that the hepatocytes were coping with excessive lipids by upregulating relevant metabolic genes and pathways. However, the boosted depletion of lipids was not sufficient to consume the overload of fatty acids, which were mainly originated from adipose tissues and transported into hepatocytes by CD36.

### Casual effects of GH on the development of NAFLD

GWAS analyses have accumulated numerous studies associated with NAFLD. There are 221 single-nucleotide polymorphisms (SNPs) and 30 studies associated with NAFLD (EFO_0003095) in the GWAS Catalog (updated to 2023.06.18). The genes mapped to the SNPs are involved in the development of NAFLD, such as *PNPLA3* [49, 50], leptin receptor (*LEPR*) [49], and human leukocyte antigen (*HLA*) [51]. We extracted the gene list from human NAFLD GWAS summary data and found that GH and GHR interacted with LEPR or leptin receptor overlapping transcript (LEPROT) (Supplementary Fig. S6) with String (version 11.5), which are involved in NAFLD. These results suggest that the GH levels or GHR may be causally associated with NAFLD in humans, similar to mice.

**Figure 8:**
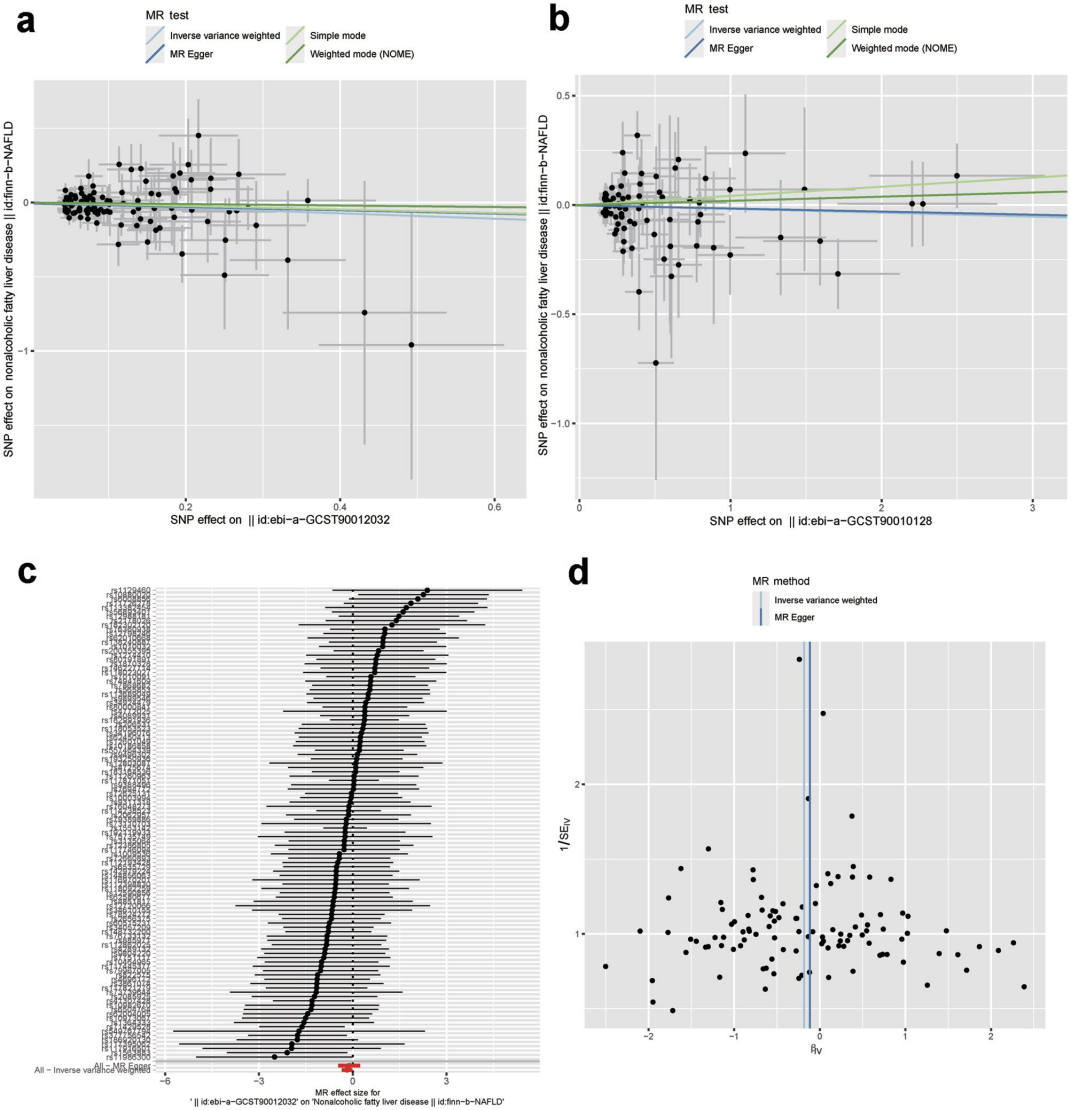
GH level has causation effects on MAFLD. (a) GH level (ebi-a-GCST90012032) (exposure) is causally related to MAFLD (finn-b-MAFLD) (outcome). *P *< 0.05. (b) GH level (ebi-a-GCST90010128) (exposure) is not causally related to MAFLD (finn-b-MAFLD) (outcome). *P *> 0.05. (c) MR effect size for SNPs in the causal effects between GH level and MAFLD. All is under zero. (d) SNPs are uniformly distributed in the funnel plot.

To test our hypothesis, we performed two-sample MR analysis to identify the causal relationship between GH levels and NAFLD. We selected GH levels or GHR as the exposure and NAFLD traits as the outcome. The inverse-variance weighted (IVW) method, which is reported to be more powerful than others, was primarily used for the analysis. As given in Table 2, we found that only GH level (ebi-a-GCST90012032) was causally associated with MAFLD (β  <  0, *P *< 0.05), while GH level (ebi-a-GCST90010128) and GHR were not (Fig. 8a and b; Supplementary Fig. S7a and b). The negative β-value indicated that in this population, a decreased GH level may increase the risk of MAFLD (Fig. 8a and c). The SNPs were uniformly ­distributed in the funnel plot (Fig. 8d).

**Table 2:** The causal relationship between exposure and NAFLD.

id.exposure	id.outcome	Outcome	Exposure	Method	SNP number	β	se	*P*-value	Correct causal direction
prot-a-1211	finn-b-NAFLD	Nonalcoholic fatty liver disease	Growth hormone receptor	Inverse variance weighted	90	−0.01237	0.044637	0.781736	FALSE
prot-a-1211	finn-b-NAFLD	Nonalcoholic fatty liver disease	Growth hormone receptor	MR Egger	90	0.089389	0.098035	0.364364	FALSE
prot-a-1211	finn-b-NAFLD	Nonalcoholic fatty liver disease	Growth hormone receptor	Simple mode	90	−0.09305	0.158898	0.559628	FALSE
prot-a-1211	finn-b-NAFLD	Nonalcoholic fatty liver disease	Growth hormone receptor	Weighted mode (NOME)	90	−0.00277	0.144454	0.984771	FALSE
prot-c-2948_58_2	finn-b-NAFLD	Nonalcoholic fatty liver disease	Growth hormone receptor	Inverse variance weighted	18	−0.07138	0.080756	0.376766	FALSE
prot-c-2948_58_2	finn-b-NAFLD	Nonalcoholic fatty liver disease	Growth hormone receptor	MR Egger	18	−0.46893	0.337132	0.183286	FALSE
prot-c-2948_58_2	finn-b-NAFLD	Nonalcoholic fatty liver disease	Growth hormone receptor	Simple mode	18	−0.20037	0.189939	0.306217	FALSE
prot-c-2948_58_2	finn-b-NAFLD	Nonalcoholic fatty liver disease	Growth hormone receptor	Weighted mode (NOME)	18	−0.22314	0.200124	0.280369	FALSE
ebi-a-GCST90010128	finn-b-NAFLD	Nonalcoholic fatty liver disease	Growth hormone levels	Inverse variance weighted	82	−0.01777	0.023451	0.448479	FALSE
ebi-a-GCST90010128	finn-b-NAFLD	Nonalcoholic fatty liver disease	Growth hormone levels	MR Egger	82	−0.01421	0.033875	0.676012	FALSE
ebi-a-GCST90010128	finn-b-NAFLD	Nonalcoholic fatty liver disease	Growth hormone levels	Simple mode	82	0.041695	0.068249	0.542959	FALSE
ebi-a-GCST90010128	finn-b-NAFLD	Nonalcoholic fatty liver disease	Growth hormone levels	Weighted mode (NOME)	82	0.01901	0.039829	0.634445	FALSE
ebi-a-GCST90012032	finn-b-NAFLD	Nonalcoholic fatty liver disease	Growth hormone levels	Inverse variance weighted	109	−0.18143	0.087322	0.037736	TRUE
ebi-a-GCST90012032	finn-b-NAFLD	Nonalcoholic fatty liver disease	Growth hormone levels	MR Egger	109	−0.1141	0.178538	0.524122	FALSE
ebi-a-GCST90012032	finn-b-NAFLD	Nonalcoholic fatty liver disease	Growth hormone levels	Simple mode	109	−0.11682	0.28772	0.685536	FALSE
ebi-a-GCST90012032	finn-b-NAFLD	Nonalcoholic fatty liver disease	Growth hormone levels	Weighted mode (NOME)	109	−0.04999	0.229221	0.827771	FALSE
prot-b-30	finn-b-NAFLD	Nonalcoholic fatty liver disease	growth hormone 1	Inverse variance weighted	26	−0.03787	0.063838	0.552999	FALSE
prot-b-30	finn-b-NAFLD	Nonalcoholic fatty liver disease	growth hormone 1	MR Egger	26	0.140115	0.150942	0.36251	FALSE
prot-b-30	finn-b-NAFLD	Nonalcoholic fatty liver disease	growth hormone 1	Simple mode	26	0.051061	0.159758	0.751917	FALSE
prot-b-30	finn-b-NAFLD	Nonalcoholic fatty liver disease	growth hormone 1	Weighted mode (NOME)	26	0.058395	0.137087	0.673775	FALSE
finn-b-NAFLD	prot-a-1211	Growth hormone receptor	Nonalcoholic fatty liver disease	Inverse variance weighted	82	0.005483	0.011376	0.629833	FALSE
finn-b-NAFLD	prot-a-1211	Growth hormone receptor	Nonalcoholic fatty liver disease	MR Egger	82	−0.0169	0.020763	0.418108	FALSE
finn-b-NAFLD	prot-a-1211	Growth hormone receptor	Nonalcoholic fatty liver disease	Simple mode	82	−0.04843	0.038553	0.212643	FALSE
finn-b-NAFLD	prot-a-1211	Growth hormone receptor	Nonalcoholic fatty liver disease	Weighted mode (NOME)	82	−0.0171	0.024365	0.484722	FALSE
finn-b-NAFLD	prot-c-2948_58_2	Growth hormone receptor	Nonalcoholic fatty liver disease	Inverse variance weighted	15	0.055118	0.060844	0.364999	FALSE
finn-b-NAFLD	prot-c-2948_58_2	Growth hormone receptor	Nonalcoholic fatty liver disease	MR Egger	15	0.084381	0.2639	0.754241	FALSE
finn-b-NAFLD	prot-c-2948_58_2	Growth hormone receptor	Nonalcoholic fatty liver disease	Simple mode	15	−0.0882	0.107682	0.426489	FALSE
finn-b-NAFLD	prot-c-2948_58_2	Growth hormone receptor	Nonalcoholic fatty liver disease	Weighted mode (NOME)	15	−0.08314	0.102507	0.430919	FALSE
finn-b-NAFLD	ebi-a-GCST90010128	Growth hormone levels	Nonalcoholic fatty liver disease	Inverse variance weighted	76	−0.01112	0.015503	0.473267	FALSE
finn-b-NAFLD	ebi-a-GCST90010128	Growth hormone levels	Nonalcoholic fatty liver disease	MR Egger	76	−0.00716	0.02286	0.754993	FALSE
finn-b-NAFLD	ebi-a-GCST90010128	Growth hormone levels	Nonalcoholic fatty liver disease	Simple mode	76	−0.01316	0.051473	0.798979	FALSE
finn-b-NAFLD	ebi-a-GCST90010128	Growth hormone levels	Nonalcoholic fatty liver disease	Weighted mode (NOME)	76	0.000258	0.022752	0.990986	FALSE
finn-b-NAFLD	ebi-a-GCST90012032	Growth hormone levels	Nonalcoholic fatty liver disease	Inverse variance weighted	84	0.009965	0.005287	0.05947	FALSE
finn-b-NAFLD	ebi-a-GCST90012032	Growth hormone levels	Nonalcoholic fatty liver disease	MR Egger	84	0.001995	0.00869	0.818994	FALSE
finn-b-NAFLD	ebi-a-GCST90012032	Growth hormone levels	Nonalcoholic fatty liver disease	Simple mode	84	0.005358	0.018861	0.777054	FALSE
finn-b-NAFLD	ebi-a-GCST90012032	Growth hormone levels	Nonalcoholic fatty liver disease	Weighted mode (NOME)	84	0.004679	0.011325	0.680583	FALSE
finn-b-NAFLD	prot-b-30	growth hormone 1	Nonalcoholic fatty liver disease	Inverse variance weighted	33	0.026652	0.026981	0.323252	FALSE
finn-b-NAFLD	prot-b-30	growth hormone 1	Nonalcoholic fatty liver disease	MR Egger	33	0.11151	0.075343	0.148959	FALSE
finn-b-NAFLD	prot-b-30	growth hormone 1	Nonalcoholic fatty liver disease	Simple mode	33	0.050423	0.072441	0.491416	FALSE
finn-b-NAFLD	prot-b-30	growth hormone 1	Nonalcoholic fatty liver disease	Weighted mode (NOME)	33	0.088601	0.048242	0.07557	FALSE

We also conducted the reverse MR analysis between MAFLD and GH levels. However, there were no significant results indicating a causal relationship between MAFLD and GH levels or GHR, although the *P*-values for the causation relationship between MAFLD and GH levels or GHR were near 0.05 (Table 2; Supplementary Fig. S7c and d). This may be explained by the insufficient number of SNPs in GWAS analysis.

**Figure 9:**
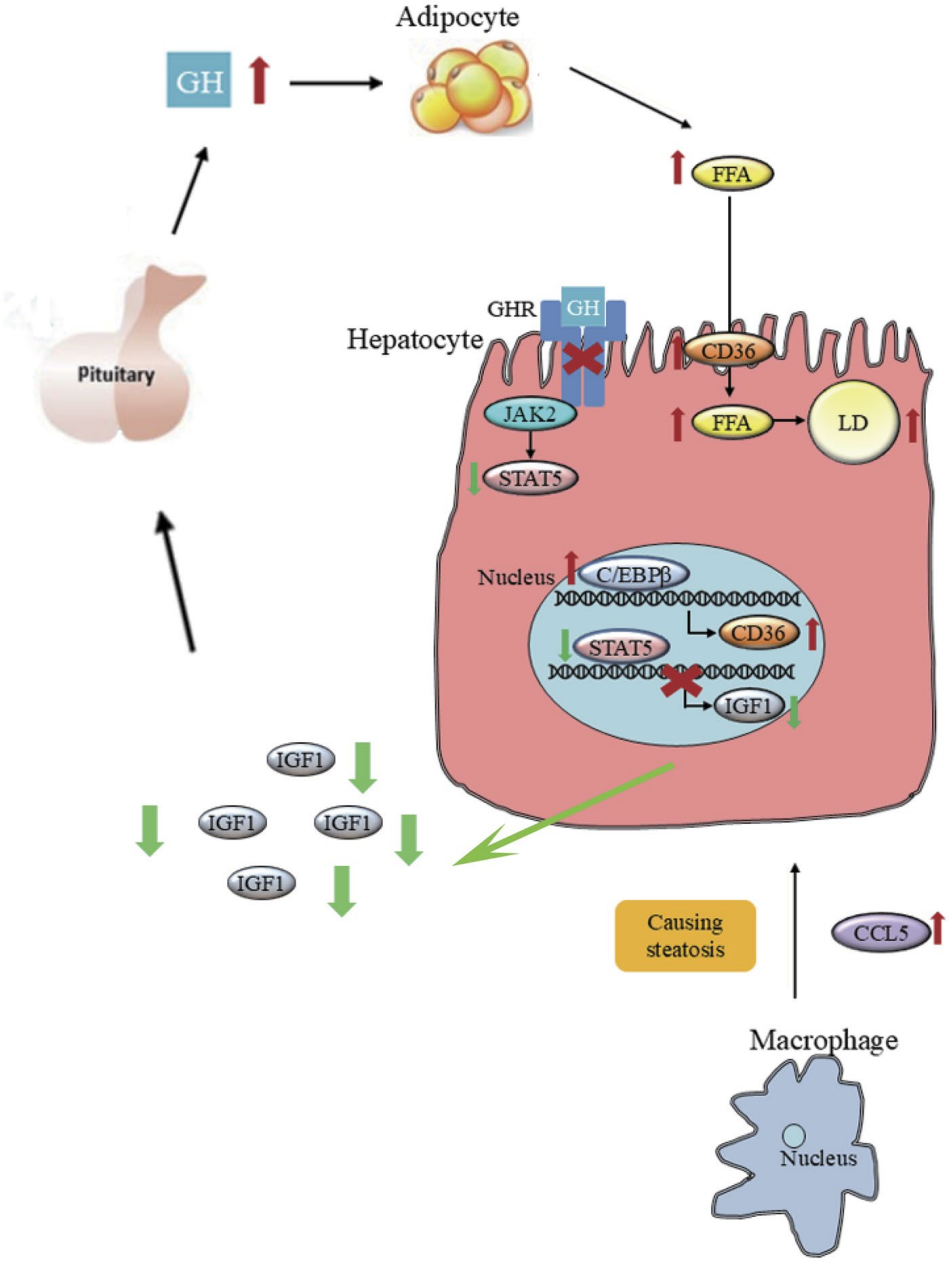
The mechanism of lean MAFLD in LGHRKO mice. Elevated GH leads to increased lipolysis and decreased lipogenesis in adipose tissues. It contributes to the reduction of fat tissues in LGHRKO mice. Besides, hydrolyzed lipid is transported into the liver in the form of FFA and increases hepatic lipid flux. Meanwhile, in the liver, hepatocytes with high levels of C/EBPβ and other lipogenic transcription factors, such as CD36, also promote *de novo* lipogenesis. The macrophage cluster expressing CCL5, which causes hepatocyte damage, is probably involved in the development of steatosis.

In summary, we speculate that: (i) reduction of IGF-1 hepatic secretion due to impaired GH signaling in LGHRKO mice leads to elevated GH synthesis; (ii) upregulated GH acting on adipose tissues causes increased lipolysis, decreased fat mass, and abundant circulating FFA; (iii)overflow FFA are transported into hepatocytes with the help of CD36; (iv) *de novo* lipogenesis, promoted by C/EBPβ and FBJ osteosarcoma oncogene (Fos), together with the extrahepatic lipids imported by CD36, collectively triggers steatosis. Meanwhile, the macrophage cluster expressing CC family chemokine is also likely involved in the development of steatosis; and (v) GH and GHR may have a similar regulatory function in the development of MAFLD in humans (Fig. 9).

## Discussion

Lean and obese MAFLD share common risks, including hyperlipi­demia, hypertension, and diabetes [52]. However, lean MAFLD patients may exhibit higher fibrosis severity and higher cardiovascular mortality, despite having similar or lower degrees of steatosis compared to obese MAFLD patients [5, 8, 53, 54]. These observations highlight the need for distinct therapeutic strategies for lean MAFLD, which are currently lacking. Existing treatments primarily target obese MAFLD or involve lifestyle intervention [6]. This therapeutic gap stems partly from the absence of suitable animal models that accurately mimic lean MAFLD pathogenesis. Current MAFLD models, such as high-fat diet (HFD) or methionine-choline-deficient (MCD) diet mice, are often reported to induce steatosis and insulin resistance alongside obesity. Although tree shrew exhibit steatosis without obesity on a high-energy diet, they do not develop insulin resistance [55], making them less representative of human lean MAFLD [56]. Here, we demonstrated that LGHRKO mice replicate key features of human lean MAFLD, including normal body weight, hyperlipidemia, hepatic steatosis, and insulin resistance, closely resembling human lean MAFLD.

Previous research has shown that hepatic GHR disruption impairs GH/IGF-1 signaling, leading to insulin resistance, glucose tolerance, and hepatic steatosis via increased lipid biogenesis and decreased hepatic lipid export [19, 57]. Fan *et al.* [19] and List *et al.* [20] both found that disruption of hepatic GHR would result in fatty liver. The body weight of GHR-deficient mice in their research is either close to or lower than control mice. Consistently, we found that LGHRKO mice have similar body weight to control mice, ­similar to the report of Cordoba-Chacon *et al.* [58], and are indeed “lean” compared to DIO mice.

As the majority of endocrine IGF-1 is originated from the liver under the mediation of GHR [59], and LGHRKO mice have hepatic GHR deficiency, it is not surprising that they have a 90% decrease in circulating IGF-I level. On the other hand, an HFD could induce the elevation of IGF-1 [60, 61], which explains the IGF-1 increase in DIO mice. In addition, IGF-1 negatively regulates the secretion of GH by targeting both the hypothalamus and pituitary [62], which explains the elevated serum GH levels in LGHRKO mice. The relationship between IGF-1 level and insulin resistance follows a U-shaped association, with both low or high IGF-1 content leading to increased homeostasis model assessment of insulin resistance (HOMA-IR, insulin resistance index) [63]. High levels of circulating GH would induce peripheral insulin resistance by its competitive binding to PI3K, uncoupling insulin receptor substrate 1 (IRS-1)-associated activation of PI3K [64]. In LGHRKO mice, low IGF-1 level and high GH level act synergistically to induce insulin resistance. This is consistent with the hypothesis that the pathogenesis of lean and obese MAFLD might be different [6].

Hyperlipoidemia is another hallmark of both lean and obese MAFLD [53]. High cholesterol, FFA, LDL, and VLDL levels, together with low HDL levels, were observed in both obese and non-obese patients [52, 65, 66]. Similar to our findings, plasma TG levels are elevated after hepatic knockdown of GHR [67]. Significant increase in VLDL and LDL was reported in the livers of 44–50-week-old GHR-deficient mice [25]. Increased plasma FFA was also found in 8-week hepatic Janus kinase 2 (JAK2) knockout mice (an intracellular tyrosine kinase binding with GHR) [21] and 12-week-old hepatic signal transducer and activator of transcription 5 (STAT5) knockout mice (a downstream transcription factor of JAK2) [68]. These pieces of evidence support dyslipidemia in LGHRKO mice.

Apart from the above similarity to DIO mice, LGHRKO mice also have reduced fat weight. The mechanism might lie in the imba­lanced GH/IGF1-1 axis. GH is known to stimulate lipolysis in adipose tissues, and increased plasma FFA in LGHRKO mice could be considered a consequence of this lipolytic activity [69]. Lack of GHR in adipocytes leads to obesity [70]. GH regulates the release of FFAs from adipocytes by modulating HSL, MGL, and ATGL [71]. On the other hand, GH also inhibits lipogenesis in adipose tissue. Similar to insulin, IGF-1 has adipogenesis effects [72]. Adipocyte knockout of IGF-1R leads to a 25% reduction of adipose tissue [73]. *Igfbp3* transgenic mice with elevated circulating IGF-1 levels (2– 3 fold) showed an 80% increase in epididymal fat pad [74]. In humans, GH increases circulating FFAs by boosting lipolysis and restraining FFA intake to adipocytes [71]. In LGHRKO mice, as the effect of increased GH and decreased IGF-1, adipose tissue releases lipids in the form of FFAs. These FFAs are absorbed into hepatocytes with the help of CD36. CCL5 is known to be secreted by many hepatic cell types, including macrophages, hematopoietic stem cells (HSCs), T cells, and so on [75]. HSC-generated CCL5 was reported to induce steatosis in hepatocytes [76]. In the case of LGHRKO mice, hepatic CCL5 is mainly generated from macrophages. We have reason to believe that macrophage-originated CCL5 is also involved in the steatosis of LGHRKO mice. In the hepatocytes of LGHRKO mice, CD36 pumps overwhelmed FFA, C/EBPβ regulates the lipogenesis process, and CCL5 in the microenvironment stimulates steatosis, which together trigger fatty liver.

Furthermore, our MR analysis suggests that GH levels may be causally associated with MAFLD in humans, supporting the translational relevance of our model. However, reverse MR analysis did not identify a significant causal relationship between MAFLD and GH/GHR, possibly due to limited SNP data. In summary, LGHKT mice represent a promising model for studying lean MAFLD. The pathogenesis involves dysregulated GH/IGF-1 signaling, adipose tissue lipolysis, hepatic FFA uptake, and macrophage-mediated inflammation. This model may facilitate a better understanding of lean MAFLD and aid in the development of targeted therapies.

### Limitations of the study

In our study, we constructed the liver GHR knockout mice and investigated the mechanism underlying the lean MAFLD caused by the loss of GHR. We revealed that loss of GHR in hepatocytes leads to attenuated insulin signaling and elevated CD36 protein level, which may contribute to the enhanced fatty acid uptake. However, the current work is subject to two major limitations. First, to directly establish causal involvement of CD36, future studies should employ GHR/CD36 double-knockout mice and perform *in vitro* FFA uptake assays using primary hepatocytes lacking both GHR and Cd36. Second, the mechanism through which hepatic GHR loss enhances adipose tissue lipolysis remains unclear; generating tissue-specific double knockout models targeting both liver and adipose GHR will be essential to dissect this inter-organ crosstalk.

## Materials and methods

### Animals

C57BL/6J mice with a pure genetic background were used in this study. LGHRKO mice were generated using the Cre/LoxP system [22] by crossing *GHR*^flox/flox^ mice [23] with *albumin-Cre* mice (B6.FVB(129)-Tg (Alb1-cre)1Dlr/J). Floxed GHR littermates served as controls. Control and LGHRKO mice were fed a regular chow diet (D12450J, 3.85 kcal/g, 10% fat calories). DIO mice were fed an HFD (D12492, 5.24 kcal/g, 60% fat calories) starting at 4 weeks of age. Mice were housed in an individually ventilated cage system with five animals per cage, maintained under 12-h light/12-h dark cycles, and provided with *ad libitum* access to food and water. Male mice aged 16 weeks were used in all experiments, with 6–9 mice per group. Mice were anesthetized prior to sacrifice. The chow diet was purchased from Keaoxieli (Beijing). All animal procedures were approved by the Animal Care and Use Committee of Dalian Medical University.

### GTT and ITT

Blood glucose concentrations were measured with glucometers (ACCU-CHEK). For the GTT, mice were fasted overnight for 16 h in cages with paper bedding. Each mouse was weighed, and glucose was administered at 2 mg/g body weight. For the ITT, mice were fasted for 4 h under the same condition. Insulin was injected at a dose of 0.75 U/kg body weight.

### Serum and liver analyses

Hormones and cytokines were measured by ELISA, including GH (BPE20916; BPRO), IGF-1 (BPE20004; BPRO), insulin (H203; Njjcbio), and CCL5 (BPE200198; ShangHai Lengton Bioscience). Serum levels of LDL cholesterol (LDL-C) (A113-1; Njjcbio), VLDL (H249; Njjcbio), total cholesterol (T-CHO) (A111-1; Njjcbio), HDL (A112-1; Njjcbio), non-esterified FFA (A042-2; Njjcbio), AST (C009-2; Njjcbio), and ALT (C010-2; Njjcbio), and hepatic contents of TG (A110-1; Njjcbio), TNF-α (SCA133Mu; USCNLIFE), Il-6 (SEA079Mu; USCNLIFE), and Il-1β (SEA563Mu; USCNLIFE) were measured according to the ­manufacturer’s instructions.

### Histology

Liver and adipose tissues were fixed in 10% formalin, processed into 5-μm paraffin sections, and stained with hematoxylin and eosin (H&E) (I032; Njjcbio). To visualize hepatic neutral lipids, fixed liver tissues were frozen, cut into 10-μm sections, and stained with Oil Red O (D027; Njjcbio). Anti-C/EBPβ (ABP53491; Abbkine) antibody was used to assess C/EBPβ expression in liver sections.

### Single-cell preparation of the liver

Mice were anesthetized via intraperitoneal injection of 1% pentobarbital (50 mg/kg). The abdomen was opened, and the portal vein was perfused with wash solution (Ca^2+^-free Hank’s balanced salt solution [HBSS] with 10 mmol/L HEPES and 0.5 mmol/L EGTA, pH 7.7–7.8). The inferior vena cava was clipped before cutting, following visible liver swollen. Wash solution was replaced with collagenase solution (dulbecco's modified eagle medium [DMEM] medium with 1 mg/mL collagenase type II) once the liver turned pale. The liver was excised once all lobes became soft. The liver was gently shaken in collagenase solution to release cells, filtered through a 40-μm filter, and centrifuged at 100 relative centrifugal force (RCF) for 5 min to collect hepatocytes. The pellet was resuspended in DMEM (containing 10% foetal bovine serum and penicillin-streptomycin), seeded in gelatin-coated plates, incubated at 37°C for 45 min, and digested with trypsin. Cell density and viability were subsequently measured.

### Single-cell RNA sequencing

Cell suspensions were loaded into Chromium microfluidic chips with 3’ chemistry and barcoded using a Chromium Controller (10X Genomics, USA). Barcoded cells underwent RNA reverse transcription, and sequencing libraries were constructed with a Chromium Single Cell 3’ v2 reagent kit (10X Genomics) according to the manufacturer’s instructions. Sequencing was performed on an Illumina HiSeq 2000. Primary cell clustering was conducted with the “cell ranger” pipeline (10X Genomics), while further analysis was performed using the “Seurat” R packages. GO and KEGG enrichment analyses were conducted with R and the “clusterProfiler” package.

### Gene expression analysis

Liver samples were stored in RNAholder^TM^ (#EH101; TransGen). Total RNA was extracted using TRIzol (#9109; Takara), reverse-transcribed using One-Step gDNA Removal and cDNA ­Synthesis SuperMix (#AT311-03; TransGen), and subjected to real-time PCR using SYBR master mix (#AQ101-03; TransGen) on an Applied Biosystems 7900HT Fast Real-Time PCR System. Transcript levels were normalized to *GAPDH*. Gene expression related to lipid breakdown and lipid synthesis were measured by quantitative PCR. The primer sequences are listed in Table 3.

**Table 3:** Primer sequence.

Target	Forward	Reverse
*GAPDH*	5′-TTGTGCAGTGCCAGCCTCGTC-3′	5′-GCGCCCAATACGGCCAAATCC-3′
*Fas*	5′-AACCTGCACTTCCACAACCC-3′	5′-GACATGAACATTGGAGCCTCCGAA-3′
*ACC1*	5′-TGCCACCACCTTATCACTATGTA-3′	5′-CCTGCCTGTCTCCATCCA-3′
*SREBP-1c*	5′-CCATCGACTACATCCGCTTC-3′	5′-GCCCTCCATAGACACATCTG-3′
*HSL*	5′-TGTGTCAGTGCCTATTCAG-3′	5′-GAACAGCGAAGTGTCTCT-3′
*ATGL*	5′-GCTGTGGAATGAGGACATAGGA-3′	5′-GCATAGTGAGTGGCTGGTGAA-3′
*MGL*	5′-ACCATGCTGTGATGCTCTCTG-3′	5′-CAAACGCCTCGGGGATAACC-3′
*PPARγ*	5′-TCGCTGATGCACTGCCTATG-3′	5′-GAGAGGTCCACAGAGCTGATT-3′

### Western blot and immunohistochemistry

Total protein was isolated from frozen liver samples stored at −80°C. Liver tissues were lysed in RIPA buffer (#PP1901; Bioteke) containing protease and phosphatase inhibitors (#ROC-5892791001 and #ROC-4906845001, respectively; Roche). Following centrifugation at 4°C, the supernatants were collected, and protein concentration was determined using the BCA assay (#P0012; Beyotime). Protein lysates (50 μg) were separated by SDS-PAGE, transferred onto nitrocellulose (NC) membranes (#66485; PALL), and blotted with the indicated primary antibodies at 1:1000 dilution: ACC1 (#A15606; ABclonal, RRID: AB_2763012), FAS (#A0461; ABclonal, RRID: AB_2757202), PPARγ (#A0270; ABclonal, RRID: AB_2757083), ATGL (#A6245; ABclonal, RRID: AB_2766852), HSL (#A15686; ABclonal, RRID: AB_2763096), MGL (#A6654; ABclonal, RRID: AB_2767241), CD36 (#A5792, Abclonal, RRID: AB_2766544), β-actin (#AC026, Abclonal, RRID: AB_2768234), and GAPDH (#10494-1-AP; Proteintech, RRID: AB_2263076).

### Two-sample MR analysis

As illustrated in Supplementary Fig. S8, SNPs from GH-related GWAS projects were selected as instrumental variables (IVs). GH-related traits were downloaded from the IEU Open GWAS project (updated to 2023.06.18). The exposures included growth hormone 1 (prot-b-30), growth hormone levels (ebi-a-GCST90012032 and ebi-a-GCST90010128), and growth hormone receptor (prot-a-1211 and prot-c-2948_58_2) (Supplementary Table S1). The outcomes were MAFLD (finn-b-NAFLD) and lean body mass (ebi-a-GCST004770) (Supplementary Tables S2 and S3). SNPs were selected based on genome wide statistical significance (*P *< 5 × 10^−5^). Linkage disequilibrium (LD) clumping was conducted under the threshold of *R*^2^ < 0.01 and clumping distance of 5000 kb. MR analysis was performed using R and the TwoSampleMR package [24], primarily based on the IVW test, along with additional analyses. Reverse MR analysis was conducted to explore the causal relationship between GH and MAFLD.

### Statistical analysis

Statistical analyses were performed using R software and associated packages. Two-tailed *t*-tests were applied to compare two groups, while one-way analysis of variance (ANOVA) followed by *post hoc* tests was used for multiple group comparisons. Results were expressed as means ± SEM, with statistical significance set at *P *< 0.05.

## Supplementary data

Supplementary data are available at *Life Metabolism* online.

## Supplementary Material

loaf037_Supplementary_Data

## Data Availability

All the data supporting the findings of this study are available within the supplementary material and corresponding authors.
