# Supplementary material for: Deletion of hepatic growth hormone receptor contributes to the pathogenesis of lean MAFLD by elevating CD36
Source: Life Metab. 2025 Oct 28;5(1):loaf037. doi: 10.1093/lifemeta/loaf037 (PMC12990309; doi:10.1093/lifemeta/loaf037)
Supplement: loaf037_Supplementary_Data [file loaf037_supplementary_data.docx]

**Supplementary Materials for**

**Deletion of hepatic growth hormone receptor contributes to the pathogenesis of lean MAFLD by elevating CD36**

Dong Yu^1,‡^, Xiao Yang^2,3,‡^, Xiaonan Zhang^1,4^, Xiaoxin Wang^1^, Zicheng Pu^1^, Ai Mi^1,6^, Liyuan Ran^1,2^, Fang Zhang ^4,*^, Bin Liang^5,*^, Yingjie Wu^1,2,*^

^1^Institute of Genome Engineered Animal Models for Human Diseases, National Center of Genetically Engineered Animal Models for International Research, Liaoning Provence Key Lab of Genome Engineered Animal Models, Dalian Medical University, Dalian, Liaoning 116044, China

^2^Shandong Provincial Hospital, Science and Technology Innovation Center，Shandong First Medical University & Shandong Academy of Medical Sciences, Jinan, Shandong 250021, China

^3^School of Life Sciences, Shandong First Medical University & Shandong Academy of Medical Sciences, Tai’an, Shandong 271016, China

^4^National Clinical Research Center for Eye Diseases, Shanghai General Hospital，Shanghai Jiao Tong University School of Medicine, Shanghai 200080, China

^5^Center for Life Sciences, School of Life Sciences, Yunnan University, Kunming, Yunnan 650091, China

^6^Jinhua Academy of Zhejiang Chinese Medical University, Jinhua, Zhejiang 310053, China

^‡^These authors contributed equally to this work.

^*^Corresponding authors. Institute of Genome Engineered Animal Models for Human Diseases, Dalian Medical University, Dalian, Liaoning 116044, China. E-mail: [yingjiewu@dmu.edu.cn](mailto:yingjiewu@dmu.edu.cn) (Y.W.); School of Life Sciences, Yunnan University, Kunming, Yunnan 650091, China. E-mail: [liangb73@ynu.edu.cn](mailto:liangb73@ynu.edu.cn) (B.L.); National Clinical Research Center for Eye Diseases, Shanghai General Hospital, Shanghai Jiao Tong University School of Medicine, Shanghai 200080, China. E-mail: [zhangfang2018@sjtu.edu.cn](mailto:zhangfang2018@sjtu.edu.cn) (F.Z.).

**
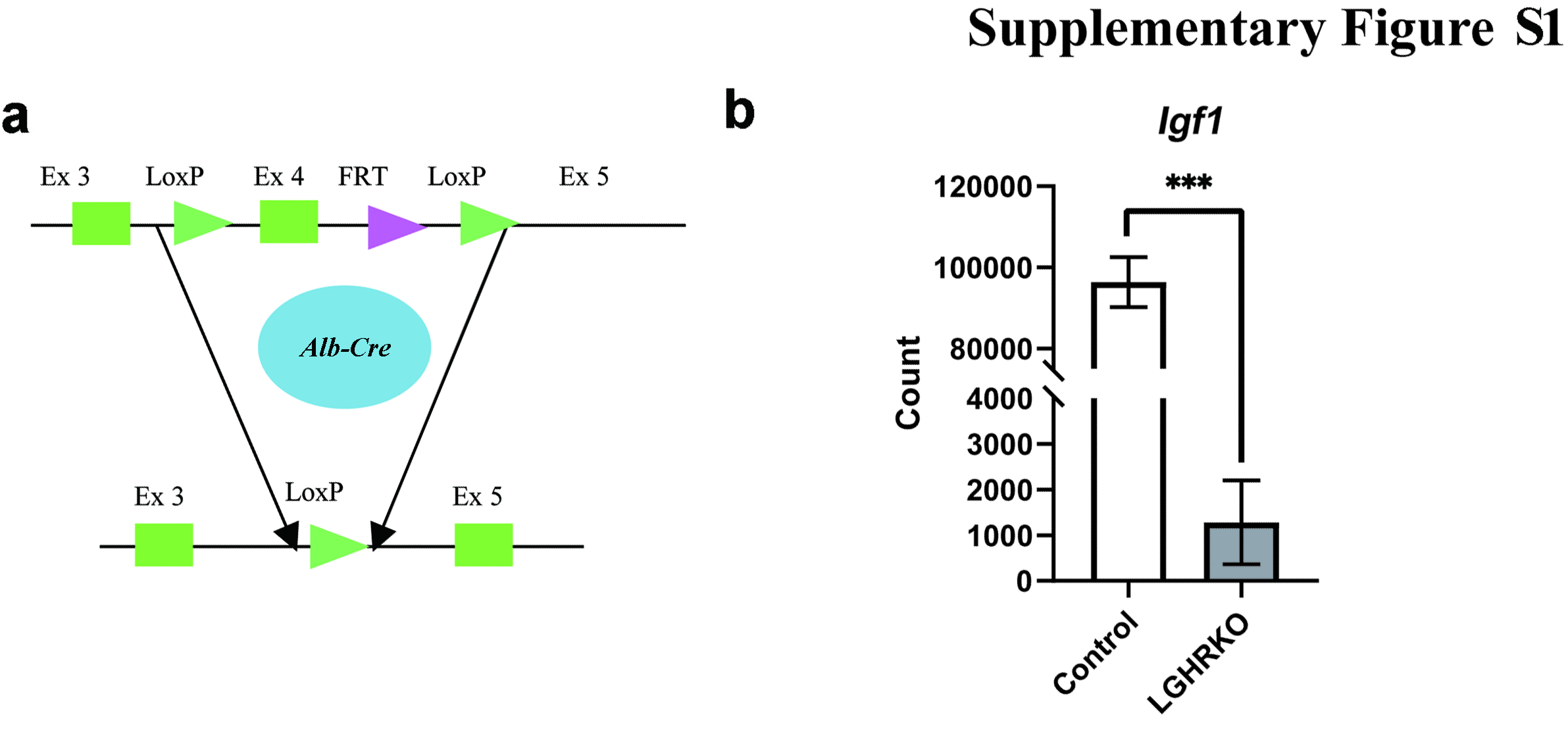
Supplementary Figure S1** Generation and validation of the LGHRKO mouse model. (a) Schematic illustration of LGHRKO mouse generation. GHR LL mice contain two LoxP sites flanking exon 4 and a flippase (Flp) recognition target (FRT) site. *Alb-cre* transgenic mice harbor an Albumin promoter-driven *Cre* transgene. (b) The expression level of *Igf1* in liver tissues of LGHRKO mice.


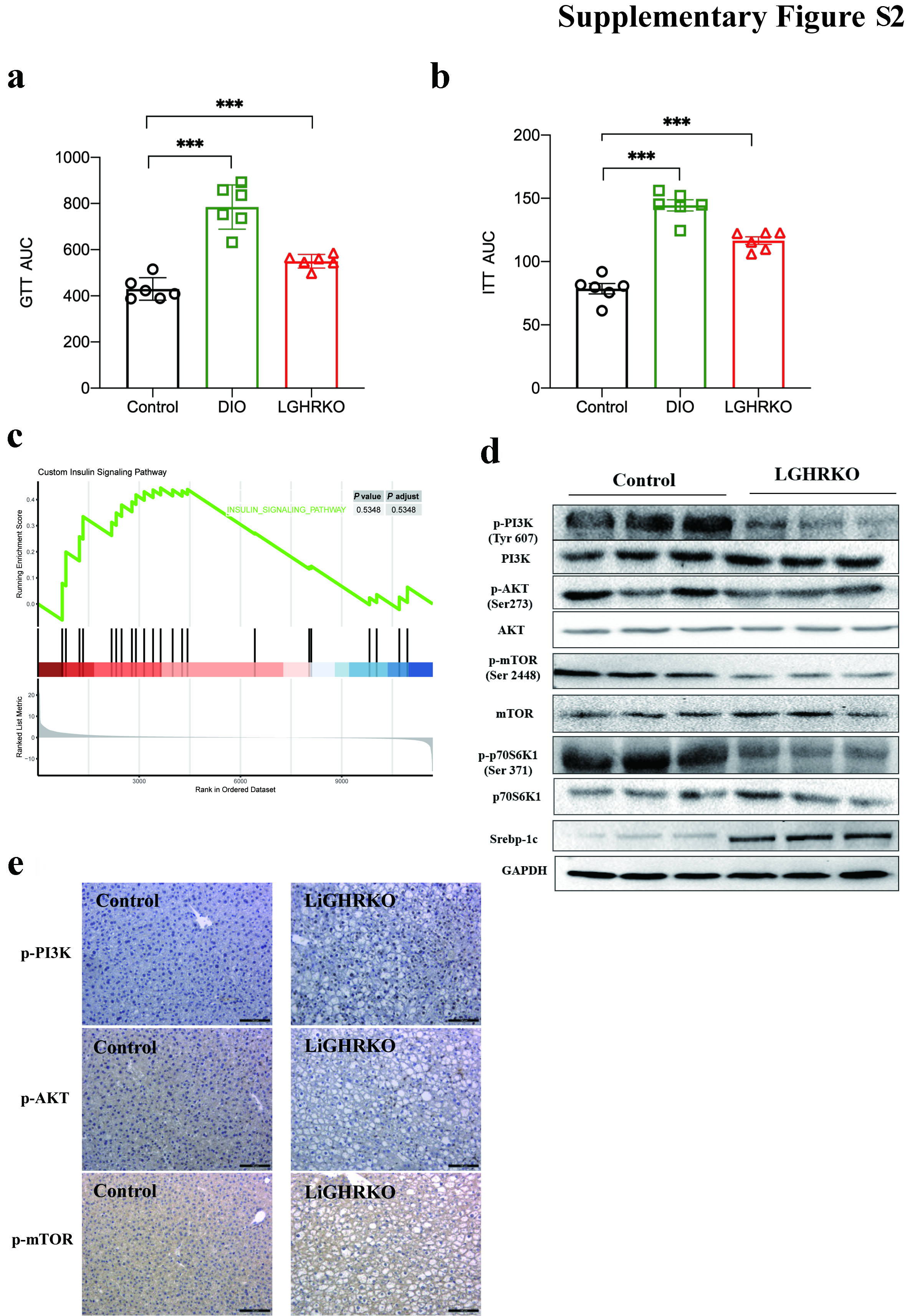


**Supplementary Figure S2** LGHRKO mice exhibit imbalanced glucose tolerance and insulin resistance. (a) The AUC of the GTT test. (b) The AUC of ITT test. (c) GSEA results of the insulin signaling pathway. (d) The phosphorylation levels of proteins in the insulin signaling pathway detected by Western blotting. (e) Immunohistochemical staining for phosphorylated proteins in insulin signaling pathway.


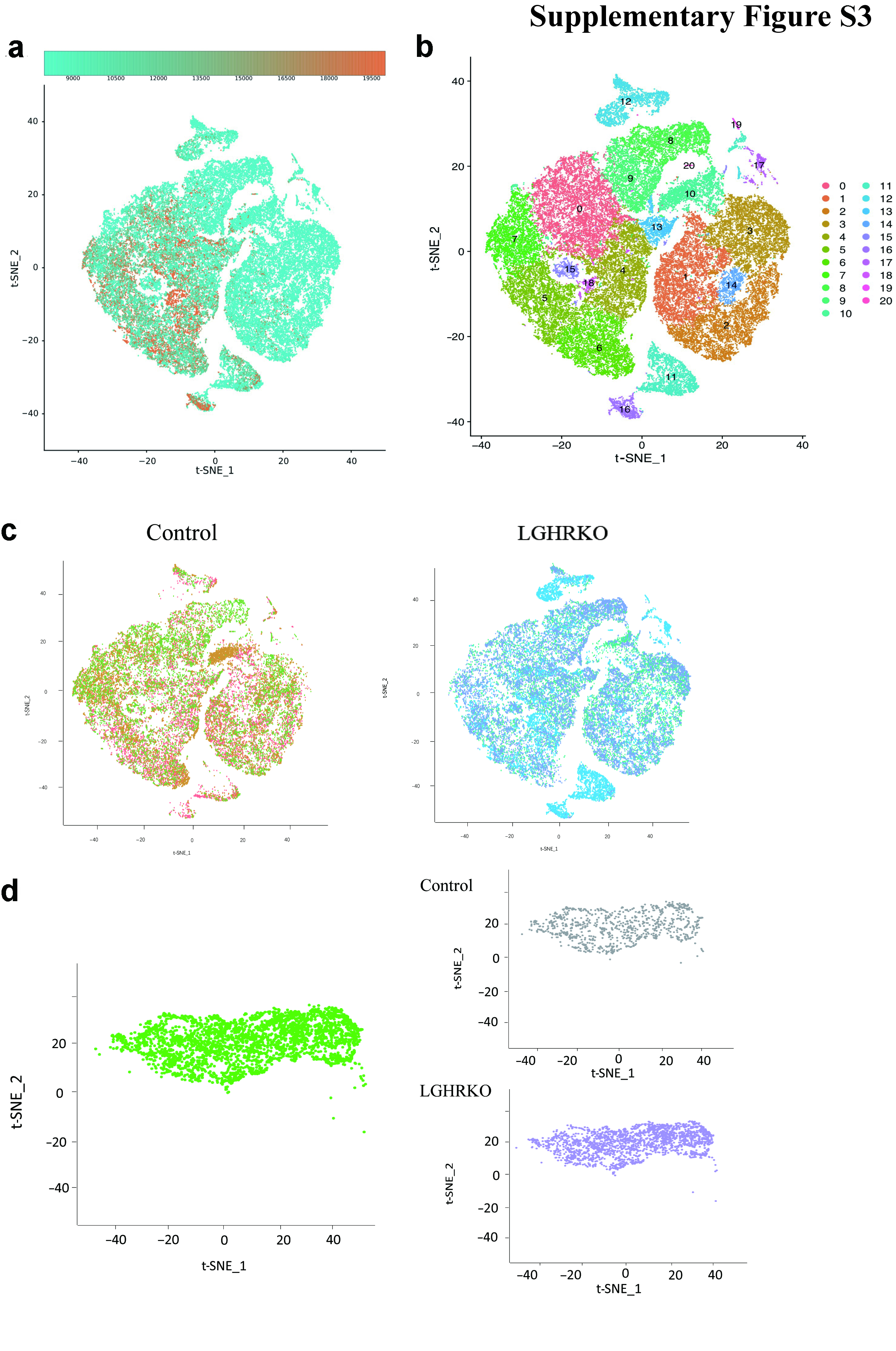


**Supplementary Figure S3** Single-cell RNA sequencing identifies distinct cell clusters in LGHRKO mice. (a) The 2D visualization of the cells presented using t-SNE. (b) A total of 66,493 cells clustered into 21 populations. (c) The visualization of the cells in control and LGHRKO mice using t-SNE. (d) t-SNE plots showing the Cluster 8.


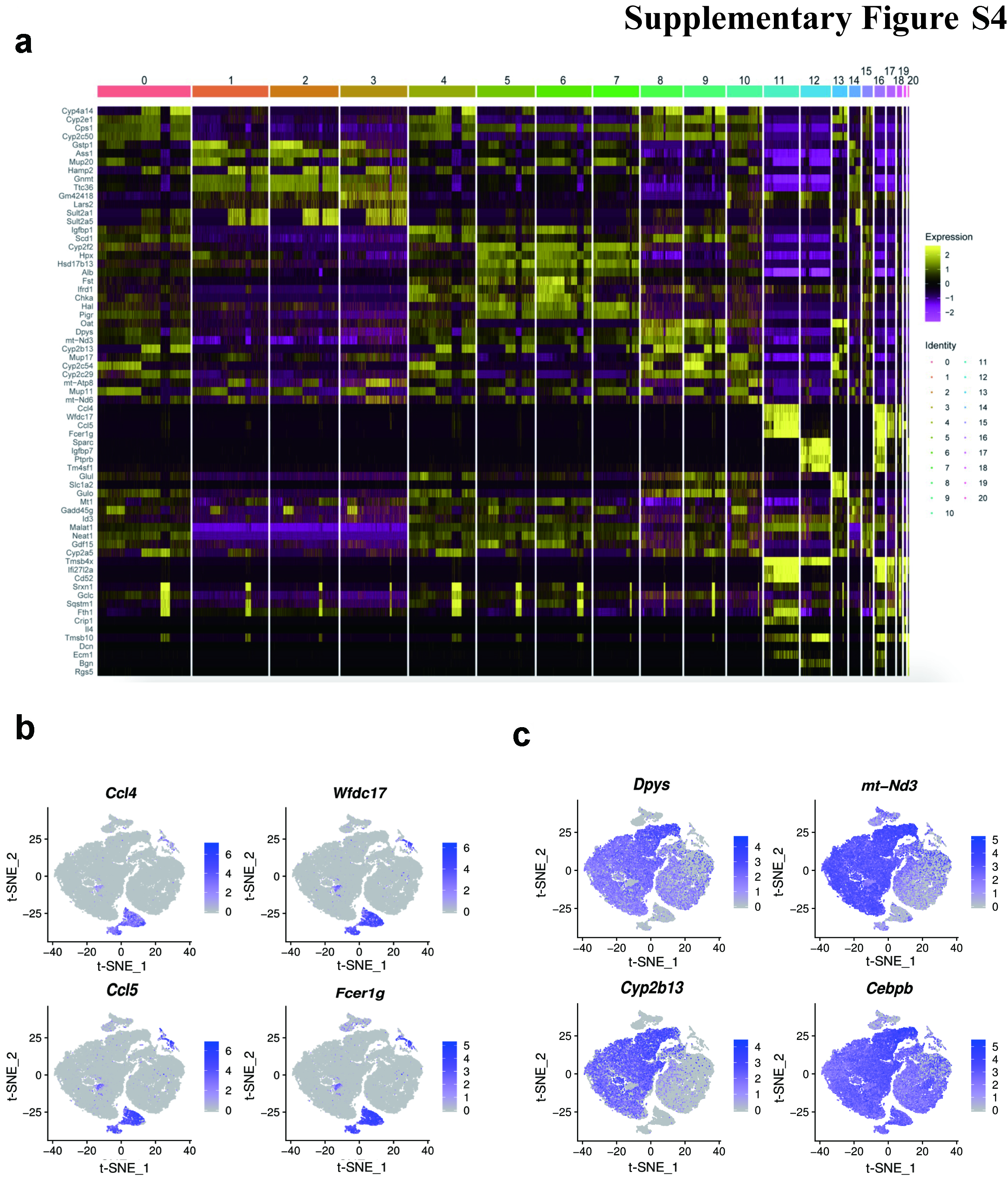


**Supplementary Figure S4** Single-cell RNA sequencing identifies distinct cell markers in the clusters of LGHRKO mice. (a) Heatmap depicting highly expressed genes among liver cell clusters. (b) t-SNE plots showing the feature genes of Cluster 11. (c) t-SNE plots showing the feature genes of hepatocytes.


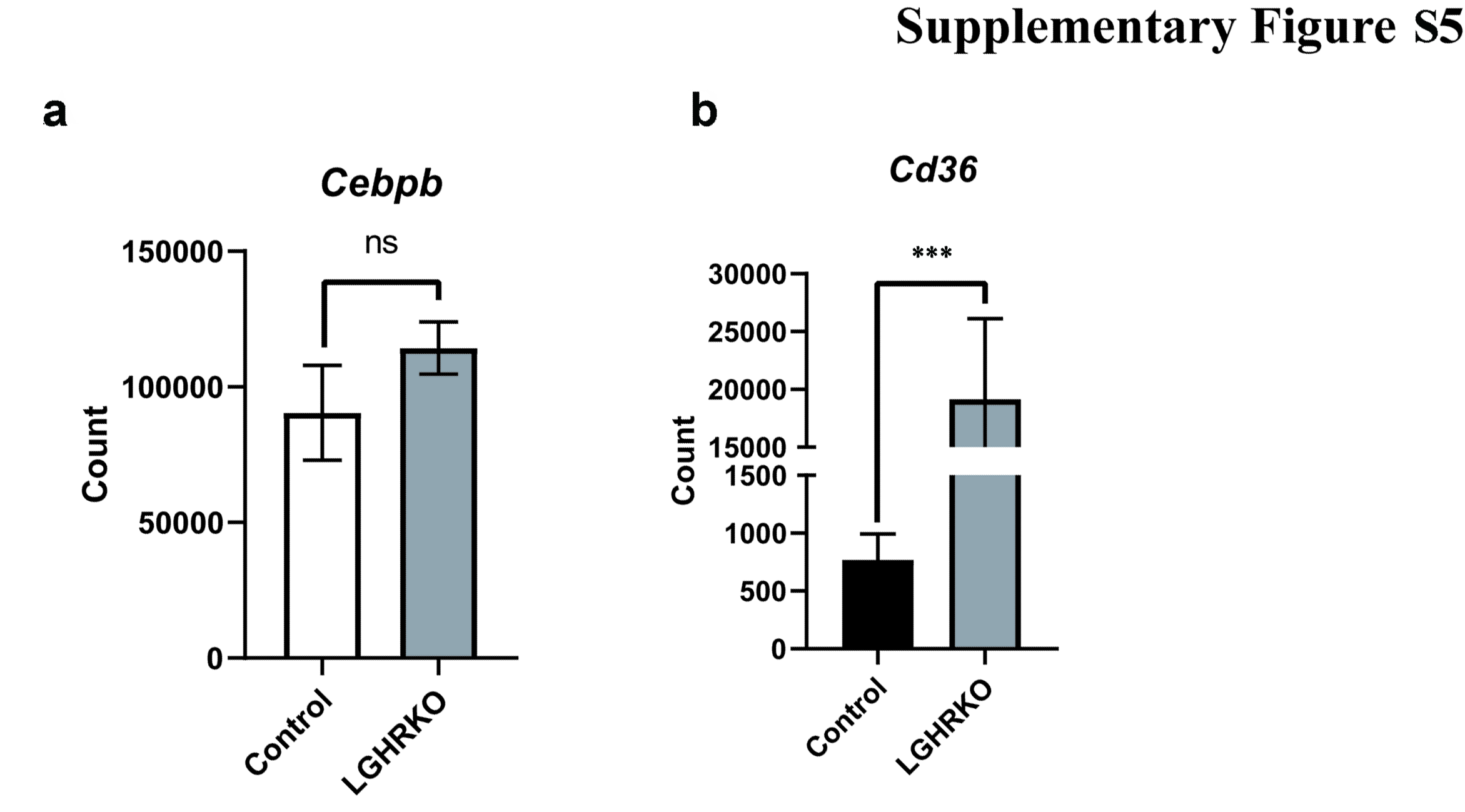


**Supplementary Figure S5** Expression analysis of genes related to lipid transport in LGHRKO mouse livers. (a) Expression level of *Cebpb* in control and LGHRKO mouse livers. (b) Expression level of *Cd36* in control and LGHRKO mouse livers.


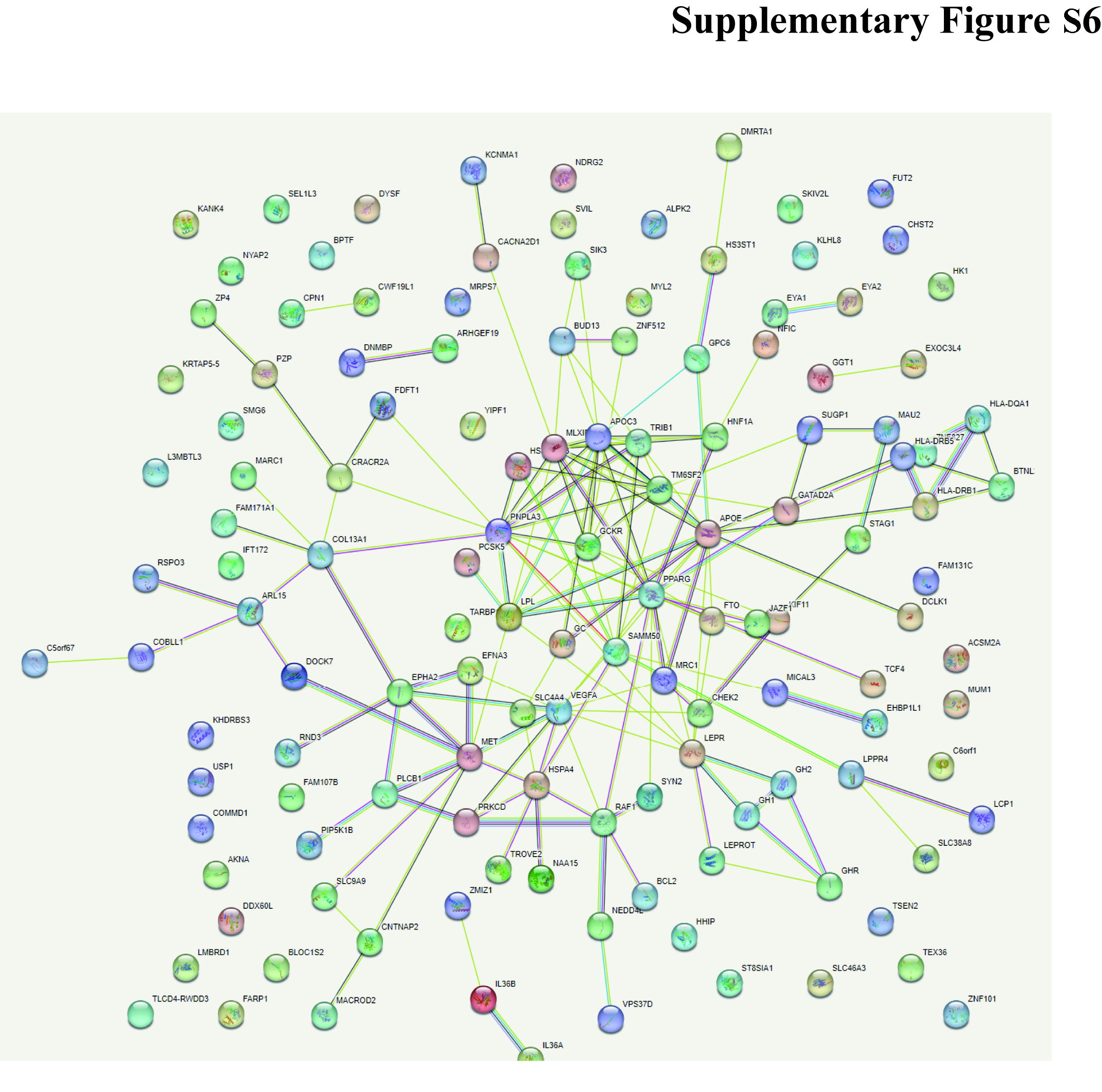


**Supplementary Figure S6** Protein-protein interaction (PPI) analysis of genes associated with NAFLD. GH and GHR interacted with LEPR or LEPROT.


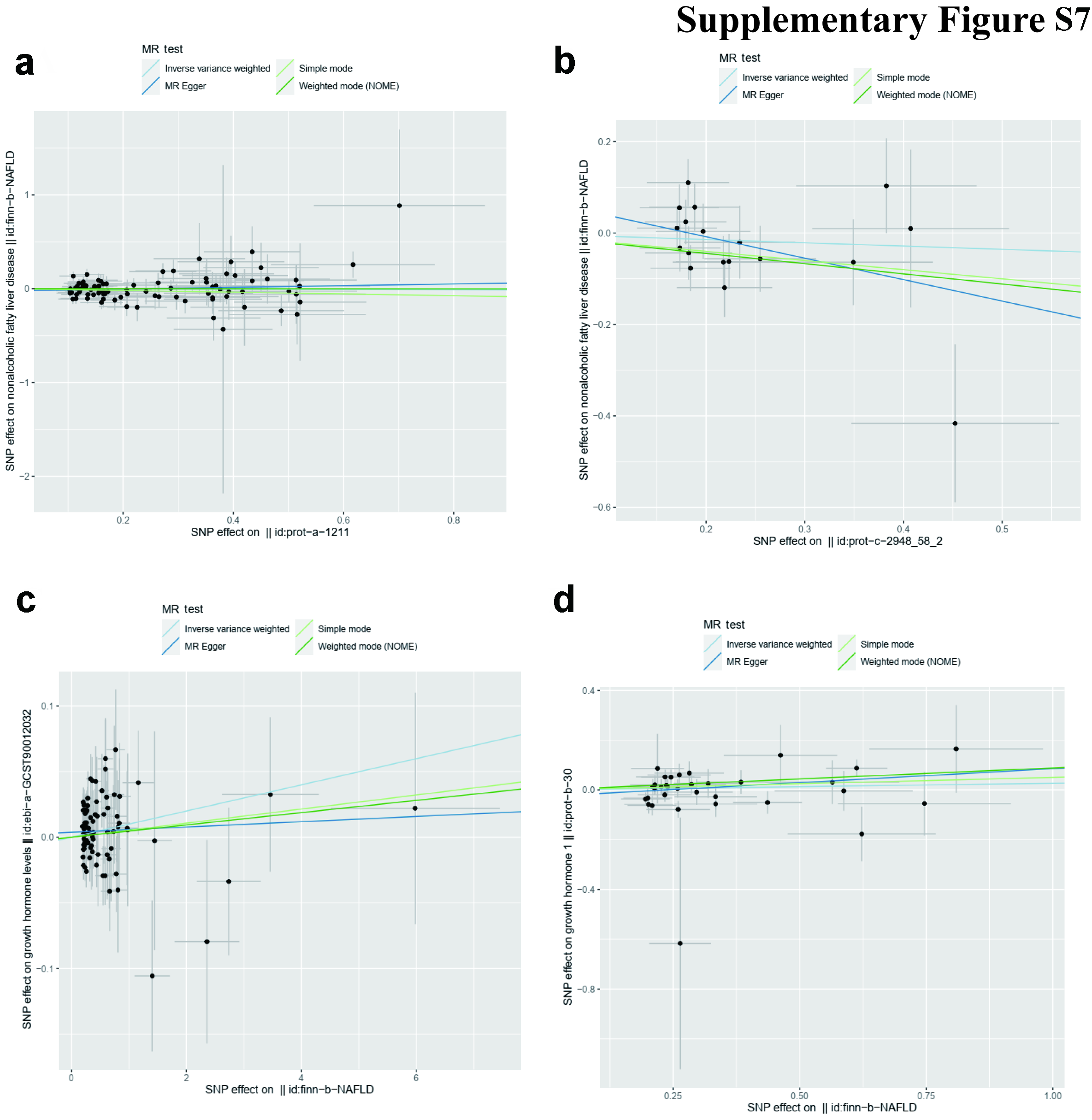


**Supplementary Figure S7** Reverse MR analysis between MAFLD and GH levels. (a−d) There are no significant results indicating a causal relationship between MAFLD and GH or GHR.


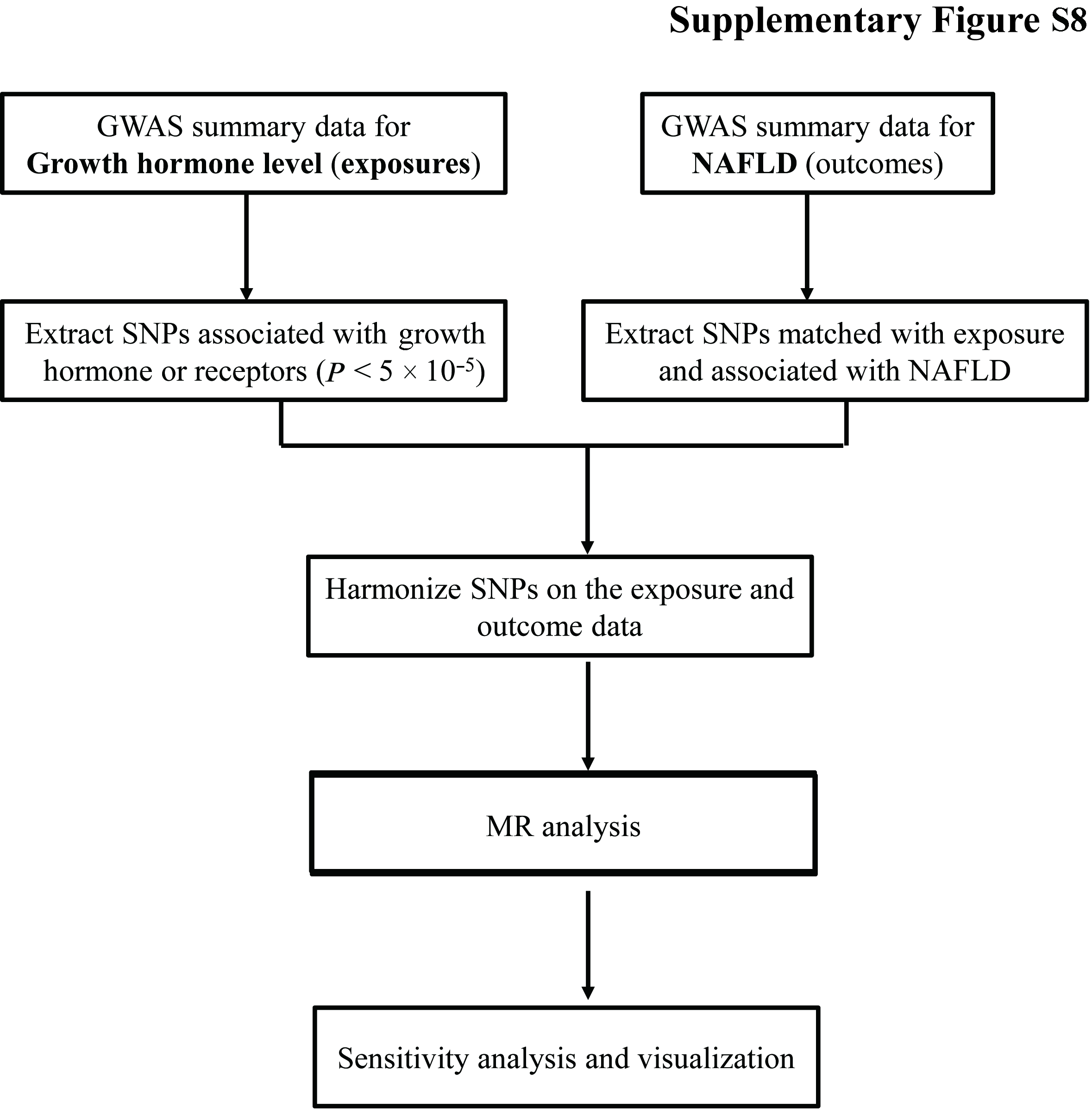


**Supplementary Figure S8** Schematic overview of MR analysis procedure. A graphic depiction of the workflow used for MR analysis.

| **Supplementary Table S1** Growth hormone related GWAS data summary. | | | | | |
| --- | --- | --- | --- | --- | --- |
| GWAS ID | Year | Trait | Consortium | Sample size | Number of SNPs |
| ebi-a-GCST90012032 | 2020 | Growth hormone levels | NA | 21,758 | 13,102,264 |
| prot-b-30 | 2018 | Growth hormone 1 | NA | 3,394 | 5,270,646 |
| prot-a-1211 | 2018 | Growth hormone receptor | NA | 3,301 | 10,534,735 |
| ebi-a-GCST90010128 | 2020 | Growth hormone levels | NA | 1,301 | 18,166,693 |
| prot-c-2948_58_2 | 2019 | Growth hormone receptor | NA | — | 501,428 |

| **Supplementary Table S2** NAFLD related GWAS data summary. | | | | | |
| --- | --- | --- | --- | --- | --- |
| GWAS ID | Year | Trait | Consortium | Sample size | Number of SNPs |
| finn-b-NAFLD | 2021 | Nonalcoholic fatty liver disease | NA | — | 16,380,466 |

| **Supplementary** **Table S3** Lean body mass related GWAS data summary. | | | | | |
| --- | --- | --- | --- | --- | --- |
| GWAS ID | Year | Trait | Consortium | Sample size | Number of SNPs |
| ebi-a-GCST004770 | 2017 | Lean body mass | NA | 8,327 | 2,272,477 |
